# Insulin-incubated palladium clusters promote recovery after brain injury

**DOI:** 10.1186/s12951-022-01495-6

**Published:** 2022-06-25

**Authors:** Shengyang Fu, Shu Zhao, Huili Chen, Weitao Yang, Xiaohuan Xia, Xiaonan Xu, Zhanping Liang, Xuanran Feng, Zhuo Wang, Pu Ai, Lu Ding, Qingyuan Cai, Yi Wang, Yanyan Zhang, Jie Zhu, Bingbo Zhang, Jialin C. Zheng

**Affiliations:** 1 Center for Translational Neurodegeneration and Regenerative Therapy, Tongji Hospital, Tongji University School of Medicine, Shanghai, 200065 China; 2 The Institute for Translational Nanomedicine, Shanghai East Hospital, Shanghai, 200120 China; 3 The Institute for Biomedical Engineering & Nano Science, School of Medicine, Tongji University, Shanghai, 200092 China; 4 Shanghai Frontiers Science Center of Nanocatalytic Medicine, Tongji University School of Medicine, Shanghai, 200331 China; 5 Translational Research Institute of Brain and Brain-like Intelligence, Shanghai Fourth People’s Hospital affiliated to Tongji University School of Medicine, Shanghai, 200434 China; 6 Key Laboratory of Spine and Spinal cord Injury Repair and Regeneration (Tongji University), Ministry of Education, Shanghai, 200065 China; 7 Wuxi Clinical College of Anhui Medical University, Hefei, 230022 China; 8 Franklin & Marshall College, Lancaster, PA 17603 United States; 9 Center for Translational Neurodegeneration and Regenerative Therapy, Yangzhi Rehabilitation Hospital affiliated to Tongji University, Shanghai, 200065 China; 10 Center for Translational Neurodegeneration and Regenerative Therapy, Shanghai Tenth People’s Hospital, Tongji University School of Medicine, Shanghai, 200072 China

**Keywords:** Traumatic brain injury, Reactive oxygen species, Biomimetic synthesis, Palladium cluster, Insulin

## Abstract

Traumatic brain injury (TBI) is a cause of disability and death worldwide, but there are currently no specific treatments for this condition. Release of excess reactive oxygen species (ROS) in the injured brain leads to a series of pathological changes; thus, eliminating ROS could be a potential therapeutic strategy. Herein, we synthesized insulin-incubated ultrasmall palladium (Pd@insulin) clusters via green biomimetic chemistry. The Pd@insulin clusters, which were 3.2 nm in diameter, exhibited marked multiple ROS-scavenging ability testified by the theoretical calculation. Pd@insulin could be rapidly excreted via kidney-urine metabolism and induce negligible adverse effects after a long-time treatment in vivo. In a TBI mouse model, intravenously injected Pd@insulin clusters aggregated in the injured cortex, effectively suppressed excessive ROS production, and significantly rescued motor function, cognition and spatial memory. We found that the positive therapeutic effects of the Pd@insulin clusters were mainly attributed to their ROS-scavenging ability, as they inhibited excessive neuroinflammation, reduced cell apoptosis, and prevented neuronal loss. Therefore, the ability of Pd@insulin clusters to effectively eliminate ROS, as well as their simple structure, easy synthesis, low toxicity, and rapid metabolism may facilitate their clinical translation for TBI treatment.

**Supplementary Information:**

The online version contains supplementary material available at 10.1186/s12951-022-01495-6.

## Introduction

Traumatic brain injury (TBI), which can be caused by traffic accidents, falls or violence, is a significant health issue that affects more than 50 million people each year [1]. Currently, debridement is the main treatment for TBI and there are no specific drugs [2]. The pathogenesis of TBI is complex. Primary injury, which is accompanied by cell death, occurs acutely as a result of the external impact. Secondary injury, for which effective treatments are lacking, involves a series of long-term cascade reactions [3, 4]. Among these cascade reactions, excessive production of reactive oxygen species (ROS), including superoxide anion, hydrogen peroxide, hydroxyl radical, singlet oxygen, etc. is considered central to inducing proinflammatory cytokine release and neuronal loss, which ultimately leads to disability or death [5]. Therefore, reducing ROS levels is considered as a promising therapeutic strategy for TBI.

Generally, there are two kinds of ROS scavengers, namely, natural enzymes and antioxidant drugs [6]. An increasing number of enzymes are being discovered, and their suitability for biomedical applications has been improved; however, these enzymes are not suitable for TBI treatment because they cannot scavenge multiple types of ROS. Several types of ROS rather than a single type of ROS involve in TBI; thus, multiple enzymes and tailored delivery systems should be used. Moreover, enzymes are often expensive, have low stability, and are difficult to recycle. In contrast, antioxidants are usually stable and cost-effective, but their specificity and effective duration are not as good as those of enzymes [7].

In recent decades, nanozymes, which are nanomaterials with enzymatic properties, have been discovered and synthesized. Nanozymes are more stable, more durable and cheaper than natural enzymes [8]. One class of nanozymes that has attracted attention are those that can mimic the catalytic properties of peroxidase, catalase and superoxide dismutase [9]. These nanozymes have been found to be effective in scavenging ROS [10], and their outstanding stability is the result of their components, which are usually metal oxides or noble metals (gold, platinum, palladium (Pd), etc.). The ROS-scavenging ability of these nanozymes is long-lasting because it involves cyclic catalytic reactions without performance loss, that is superior to the molecular drugs [10]. Notably, some nanozymes, such as thioether-functionalized nanoparticles (16.4 nm) [11], lipoprotein nanoparticles (66.5 nm) [12], and Au nanoclusters (2 nm) [13], have achieved good results in TBI mice. However, these nanozymes still need to be improved, for example, simplifying their synthesis processes, further reducing their size, enhancing their therapeutic efficacy, and modifying them for clinical application [14].

**Fig. 1:**
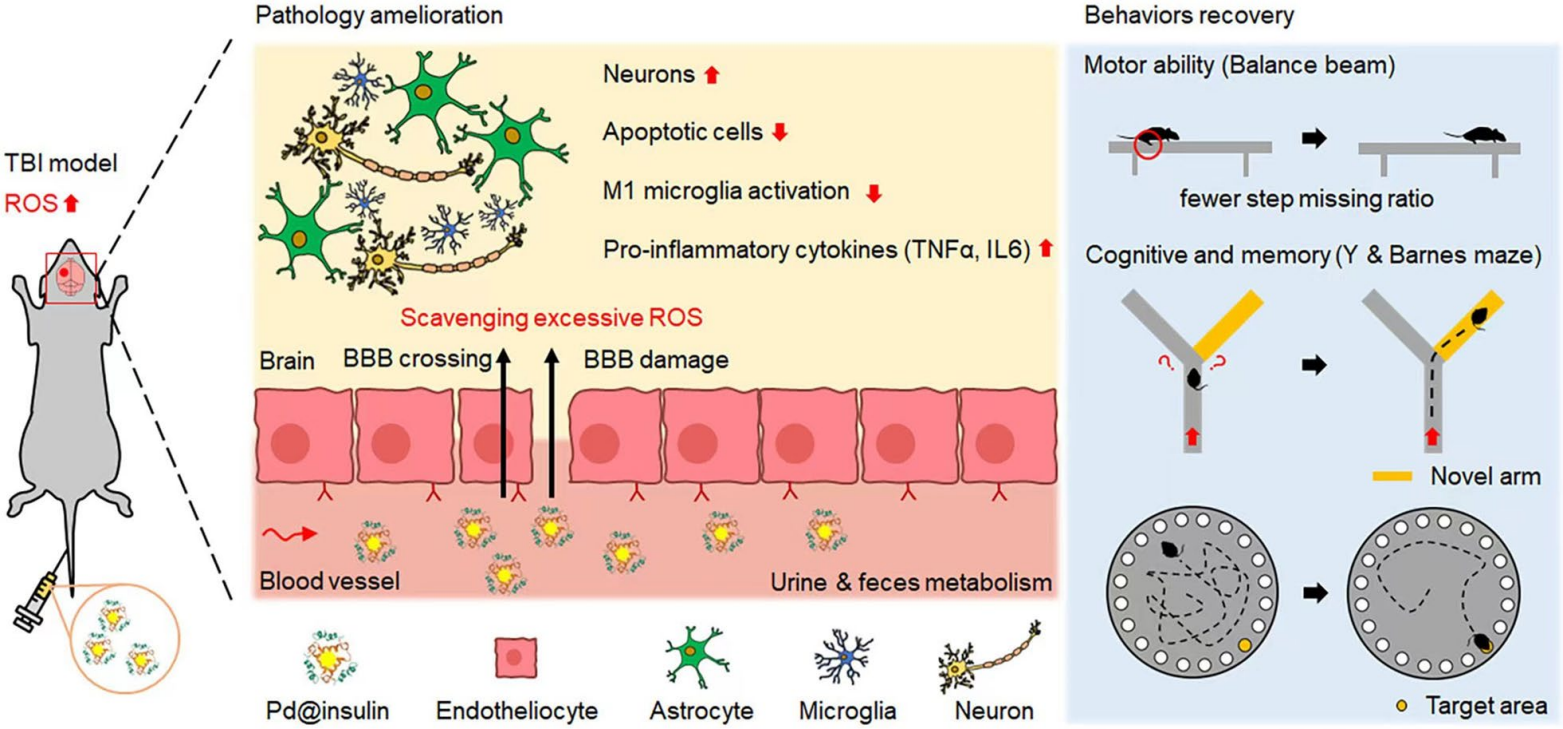
Scheme illustration. Pd@insulin nanoclusters scavenge excessive multiple ROS, ameliorate a series of negative pathologies, and TBI-induced recovery motor, function, learning, and spatial memory impairment

Here, we designed a novel biomimetic synthesis method for preparing ultrasmall Pd nanoclusters. The synthesis principle leverages protein-mediated biomimetic biomineralization and the spatial confinement effect [15]. Unlike traditional chemical methods, this strategy is convenient, green, and highly effective without a further modification. We chose Pd as a nanozyme because it is able to mimic superoxide dismutase (SOD) and catalase (CAT) and scavenges hydroxyl radical, and thus may effectively reduce ROS in the context of TBI [16]. Insulin was selected as the template because it exerts a confinement effect in the synthesis of ultrasmall nanoparticles, has a positive therapeutic effect on TBI at low doses, and can cross the blood-brain-barrier (BBB) with nanoparticles via receptor-mediated transcytosis which may facilitate the BBB penetration of nanoparticles [17, 18]. For the first time, we successfully synthesized insulin-incubated ultrasmall Pd clusters (Pd@insulin), which exhibited excellent multiple ROS-scavenging ability in the brains of TBI mice and ameliorated TBI-induced motor function, learning, and spatial memory impairment. Given that this process is biomimetic, scalable, and does not involve any complicated processes or further modification, it has great application potential in the clinic and may inspire the development of other nanodrugs for the treatment of TBI or other ROS-related diseases (Fig. 1).

## Results

### Insulin-incubated ultrasmall palladium cluster synthesis and characterization

**Fig. 2:**
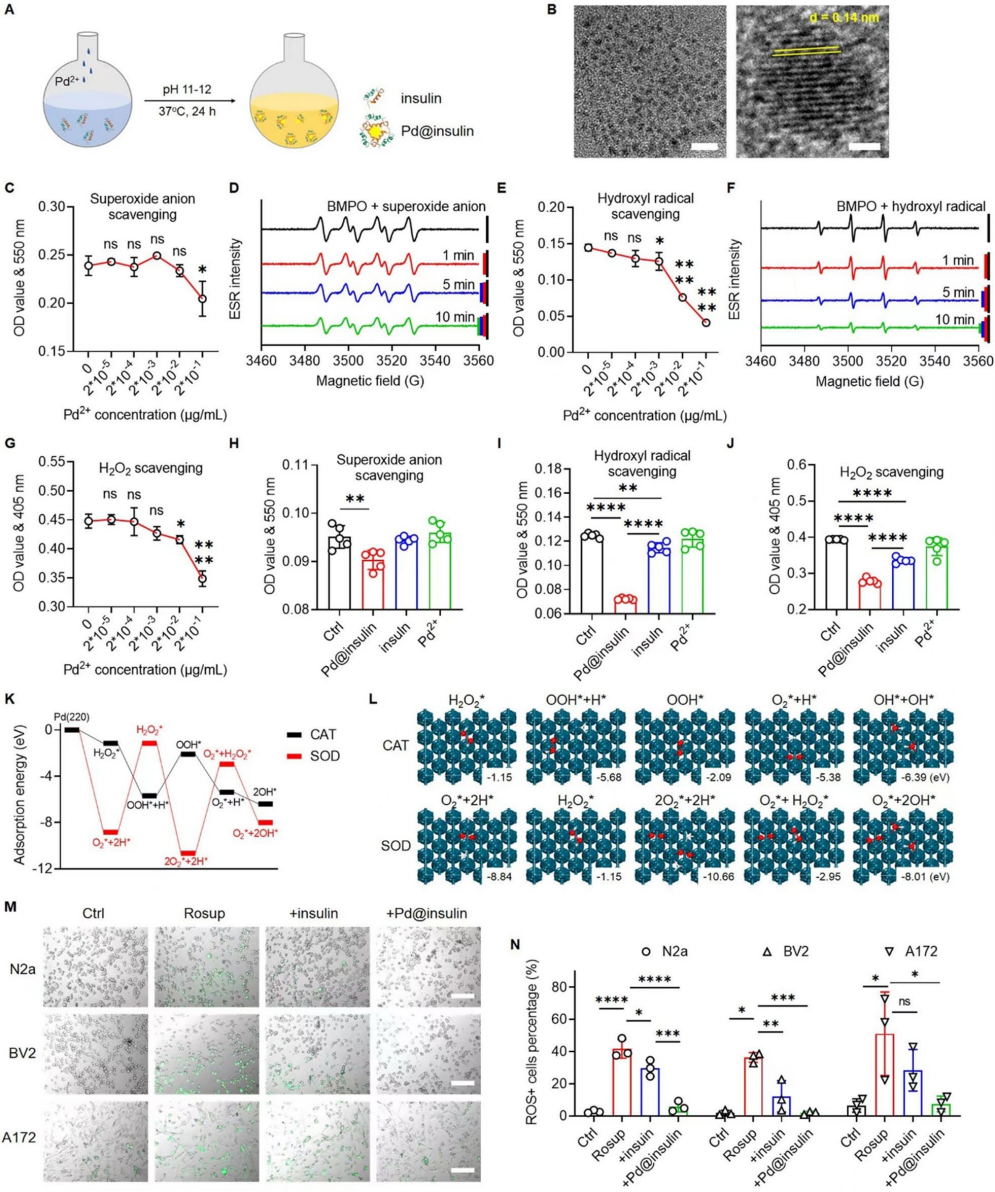
Characterizations and multiple ROS scavenging ability of Pd@insulin clusters. **A** Scheme illustration of Pd@insulin synthesis via the protein biomimetic method. **B** HRTEM images of Pd@insulin (Scale bar: 10 nm left, 1 nm right). **C** Superoxide anion **E** hydroxyl radical, and **G** H_2_O_2_ scavenging ability of Pd@insulin (n = 4). Time depended **D** superoxide anion and **F** hydroxyl radical scavenging ability of Pd@insulin by EPR spectrum. Comparison of **H** superoxide anion **I** hydroxyl radical **J** H_2_O_2_ scavenging ability between Pd@insulin cluster, insulin, and Pd^2+^ (n = 5). **K** Adsorption energies profiles of catalytic process of the SOD and CAT processes for Pd, and **L **geometry structures of the intermediate states (red and white dots represent oxygen and hydrogen atoms respectively; * represents absorption state). **M** Intracellular ROS scavenging ability evaluation via N2a, BV2, and A172 cells (Rosup is positive control, green fluorescence represents ROS positive) (Scale bar 200 μm), and **N** corresponded quantitative results (n = 3). Data are all shown as mean ± SD. Statistical analysis of (**C**, **E**, **G**, **H**, **I**, **J,** and **N**) was performed by one-way ANOVA with a Tukey post hoc test

Pd@insulin clusters were synthesized via a green and “one-pot” biomimetic method using insulin as a template (Fig. 2A). Pd^2+^ ions pre-anchored by insulin were reduced into elemental Pd in situ under alkaline conditions. This synthesis process is convenient, environmentally benign, and can be reproduced to allow large-scale synthesis. To obtain a high-quality product, the effect of the ratio of Pd^2+^ to insulin on the formation of clusters was explored (Additional file 1: Table S1). Optimization of this ratio allowed the synthesis of uniform nanoclusters, while insufficient or excess insulin resulted in aggregation (Additional file 1: Fig. S1). When the optimized ratio and mild reaction conditions (37 °C, 24 h, pH = 11–12) were used, the Pd@insulin clusters were homogeneously distributed nanospheres with a clear crystal lattice structure and an average diameter of 3.2 nm (Fig. 2B, Additional file 1: Fig. S2), which benefits in vivo accumulation and clearance [19]. Compared with traditional chemical synthesis processes, protein-incubated strategies are conducive to incubating small and monodispersed nanoparticles because proteins serve as reaction site providers and stabilizers to inhibit particle overgrowth and prevent aggregation [15].

To confirm Pd cluster formation, XPS was performed for qualitative element analysis. The results demonstrated the existence of Pd, including Pd^2+^ and Pd^0^, based on the Pd^0^ 3d5/2 (335.63 eV) and Pd^0^ 3d3/2 (341.08 eV) peaks and Pd^2+^ 3d5/2 (336.89 eV) and Pd^2+^ 3d3/2 (342.23 eV) peaks (Additional file 1: Fig. S3A). The existence of Pd^0^ proved that clusters formed, while Pd^2+^ was mainly a residual precursor absorbed by insulin [20].

In this study, insulin served not only as a template for Pd cluster formation but also to protect against and treat TBI pathology. CD spectroscopy was further used to study the structural change in insulin before and after the chemical reaction. Our results showed that Pd@insulin possessed α-helix but that the structure was slightly changed (Figure S3B) due to the combined effect of alkali/Pd cluster formation-induced protein structure changes [21]. TGA was used to calculate the weight ratio of Pd clusters in Pd@insulin clusters. We found that Pd clusters accounts for 8% of the weight of Pd@insulin clusters (Additional file 1: Fig. S3C). Interestingly, Pd@insulin clusters can be made into a powder by freeze-drying. SEM revealed that the Pd@insulin cluster powder comprised O, C, S, N, and Pd (Additional file 1: Fig. S4). After being redissolved in water, there was no significant variation in hydrodynamic size or ultraviolet-vis absorption spectra, indicating good stability (Additional file 1: Figs. S5–7).

Collectively, the above experimental results demonstrate that ultrasmall Pd@insulin clusters were formed via the protein incubation strategy. Our results show that this strategy is facile, green, and effective and that the obtained Pd clusters are naturally coated with insulin without additional modification.

### Pd@insulin clusters scavenge multiple ROS

To further investigate the ROS-scavenging ability of Pd@insulin, the levels of representative ROS, i.e., superoxide anion, hydroxyl radical and H_2_O_2_, were studied. The level of superoxide anion, which is generated by the reaction between xanthine and xanthine oxidase, was quantified by measuring the absorbance at 550 nm, and a significant decrease in the superoxide anion level was observed after administration of 0.2 µg/mL Pd^2+^ (Fig. 2C). The level of hydroxyl radical, which is generated by the Fenton reaction, was similarly quantified, and Pd@insulin began consuming hydroxyl radical to a significant degree when the Pd^2+^ concentration reached 2*10^− 3^ µg/mL (Fig. 2E). H_2_O_2_ consumption was evaluated by reacting H_2_O_2_ with ammonium molybdate and measuring the absorbance of the resulting yellow solution at a wavelength of 405 nm. Pd@insulin was able to scavenge H_2_O_2_ beginning when the Pd^2+^ concentration reached 0.02 µg/mL (Fig. 2G), and the consumed H_2_O_2_ did not convert into other types of free radicals (Additional file 1: Fig. S8). Pd@insulin was also proven to have superoxide anion- and hydroxyl radical-scavenging ability via EPR analysis (Fig. 2D, F). The results indicated the scavenging ability of Pd@insulin was the best for hydroxyl radical, which has been proven to be the most reactive of the three types of ROS studied, while the superoxide anion- or H_2_O_2_-scavenging ability of Pd@insulin was consistent with the ability to mimic superoxide dismutase and catalase confirmed in other studies [16]. Furthermore, the Pd@insulin nanocluster possessed multiple rounds ROS scavenging ability without performance loss (Additional file 1: Fig. S9).

Due to the presence of residual Pd^2+^ and insulin, we further compared the ROS-scavenging ability of Pd@insulin and these raw materials (Fig. 2H–J). Insulin also possessed hydroxyl radical- and H_2_O_2_-scavenging ability, but its scavenging ability was far inferior to that of Pd@insulin. Additionally, the Pd^2+^ solution did not exhibit scavenging ability. The ability of insulin to eliminate ROS is probably related to direct protein oxidation [15]. Therefore, we regarded that Pd clusters mainly contributed to the multiple ROS-scavenging ability of Pd@insulin. To better understand the chemical mechanism for Pd@insulin-induced ROS clearage, DFT was used to calculate the adsorption energy of intermediate products and simulate the optimal adsorption mode on (220) of Pd, of which the d = 0.14 nm of interplanar crystal spacing corresponded to the crystal plane (220) of Pd (Fig. 2B, K, L). Although the ROS scavenging processes are mutually permeated and complicated, attributed to the multi-ROS scavenging ability of Pd, the total CAT-mimic reaction was simplified to the degradation of H_2_O_2_ into H_2_O and O_2_, and the SOD-mimic involves the degradation of superoxide anion into O_2_ [13]. The adsorption energies in each step of both CAT and SOD-mimics were negative, indicating the spontaneous adsorption of free radicals on Pd. In the process of CAT-mimic, H_2_O_2_ was first absorbed on Pd crystal, and decomposed into OOH* and H* states. The absorbed OOH* further decomposed into O_2_* and H* whereas disproportionation-like reaction induced another H_2_O_2_ absorption and degradation into OH* state, resulting in the release of H_2_O and O_2_. In the process of SOD mimic, superoxide anion was absorbed and decomposed into O_2_, along with the multi substance absorption states and free radicals release, which induced further adsorption or scavenged by Pd.

To evaluate the intracellular ROS-scavenging ability of Pd@insulin, Neuro-2a (N2a) cells, BV2 microglia, and A172 astrocytes were pretreated with Rosup to construct high ROS level models. The treatment time of Pd@insulin in different cells was previously studied, and it was found that most cells can take up Pd@insulin after 2 h of treatment (Additional file 1: Fig. S10). Subsequently, we found that the percentage of ROS^+^ cells (green fluorescence) was decreased by insulin or Pd@insulin in the three cell models (Fig. 2M). Specifically, Pd@insulin scavenged 92.2%, 99% and 87.8% excess intracellular ROS in N2a, BV2, and A172 cells, respectively, whereas insulin scavenged 30.7%, 75.7% and 34.14% of excess intracellular ROS, indicating that the ROS-scavenging ability of Pd@insulin was outstanding while that of insulin was limited (Fig. 2N).

### Pd@insulin clusters do not induce adverse effects

To determine the *in vitro* biocompatibility of Pd@insulin clusters, N2a, BV2, and A172 cells were treated with different concentrations of Pd@insulin and insulin (Additional file 1: Table S2). High-dose insulin was cytotoxic to BV2 and A172 cells (Additional file 1: Fig. S11), consistent with reports indicating that high concentrations of insulin dramatically suppress cell proliferation [22]. Interestingly, Pd@insulin was found to promote the proliferation of all three types in a concentration-dependent manner at both 24 and 48 h after treatment (Additional file 1: Fig. S11). Insulin naturally decorated Pd clusters formed via our efficient synthesis method, but, the toxicity of excessive insulin was inhibited after the structure was slightly changed (Additional file 1: Fig. S3B).

**Fig. 3:**
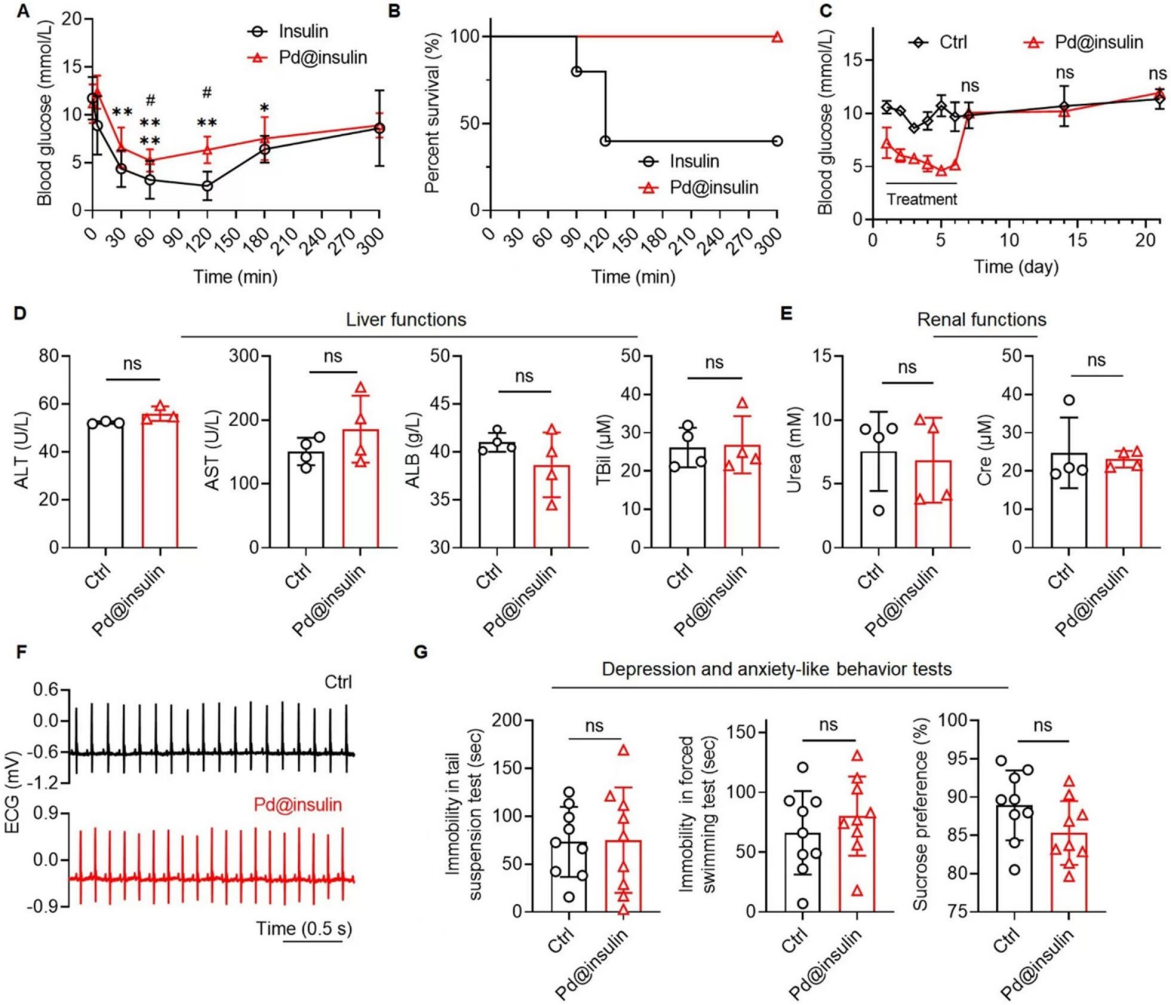
In vitro and in vivo biocompatibility assessment of Pd@insulin clusters. **A** Blood glucose variation after Pd@insulin/insulin once administration (n = 5, * represents the significant difference between each time point and initial in Pd@insulin group, # represents the significant difference between Pd@insulin and insulin at one time point). **B** Survival curve of mice after once administrated by Pd@insulin/insulin (n = 5). **C** Long term blood glucose variation during Pd@insulin daily administration for 6 days (n = 3). **D** Liver and **E** renal functions evaluation (n = 3–4). **F** Representative electrocardiographs of Pd@insulin-treated mice and normal (Ctrl) mice. **G** Depression and anxiety-like behavior tests of Pd@insulin-treated mice and normal mice (Ctrl) via TST, FST, and SPT (n = 9). Data are all shown as mean ± SD. Statistical analysis of (**A**) was performed by one-way ANOVA with a Tukey post hoc test, and **C–E** and **G** were performed by unpaired Student’s* t*-test.

To determine the appropriate concentration for *in vivo* treatment, the hemolysis ability of Pd@insulin was examined by culturing erythrocytes with Pd@insulin. Although treatment with Pd@insulin at a Pd^2+^ concentration of 20 µg/mL resulted in a hemolysis rate of 10.7%, Pd@insulin at a Pd^2+^ concentration equal to or less than 10 µg/mL resulted in a safe hemolysis rate (less than 5%) (Additional file 1: Fig. S12, Table S2) [19]. The variation in blood glucose levels after intravenous administration of Pd@insulin (Pd^2+^ concentration of 5 µg/mL, 250 µL) was examined since insulin is a crucial regulator of blood glucose levels [23]. The results showed that the blood glucose level of mice reached the minimum (from ca. 11.2 to 5.22 mmol/L) at 60 min and returned to normal levels at 180 min (Fig. 3A). The reduction in blood glucose levels after insulin administration was much more obvious than that after Pd@insulin administration, especially at 90 and 120 min (Fig. 3A), and caused some of the mice to die (Fig. 3B). The reduction in blood glucose levels induced by Pd@insulin was approximately 14% of that caused by insulin (Additional file 1: Fig. S13), which can be attributed to the insulin structure change due to the alkali-induced protein degeneration and nanoclusters growth.

To explore the feasibility of continuous treatment, Pd@insulin was intravenously administered for 6 successive days. During the administration and posttreatment phases, analysis of the variation in blood glucose levels, electrocardiography (ECG), serum biochemistry tests, histopathological analysis of major organs, and fecal examination were carried out. Although blood glucose levels were reduced 1 h after Pd@insulin administration, the long-term blood glucose level of Pd@insulin-treated mice was not significantly different from that of control mice when the treatment was terminated (Fig. 3C). Serum biochemistry parameters, including the number/percentage of leucocytes, lymphocytes, monocytes, neutrophils, and erythrocytes, the levels of hemoglobin, and hepatorenal function-related parameters (alanine transaminase, aspartate transaminase, albumin (ALB), total bilirubin (Tbil), urea and creatinine (Cre) levels) were not significantly different between Pd@insulin-injected and control mice (Fig. 3D, E, Additional file 1: Fig. S14). ECG showed that Pd@insulin had no adverse effects on the cardiac function of mice (Fig. 3F). H&E staining indicated no necrosis, congestion, or hemorrhage in the hearts, livers, spleens, lungs, kidneys, or brains of Pd@insulin-injected mice (Additional file 1: Fig. S15). The feces were negative for both occult blood and transferrin, indicating that the excretion of Pd@insulin-treated mice was normal (Additional file 1: Fig. S16).

Subsequently, the influence of Pd@insulin on emotion was evaluated. Depression- and anxiety-like behaviors were assessed by the tail suspension test (TST), forced swimming test (FST) and sucrose preference test (SPT) [24]. No significant difference in the immobility time in the TST, the immobility time in the FST, or the degree of sucrose preference was observed between Pd@insulin-injected and control mice, suggesting that Pd@insulin did not have adverse effects on emotion (Fig. 3G).

### Pd@insulin clusters cross BBB and aggregation in the injured cortex after TBI

**Fig. 4:**
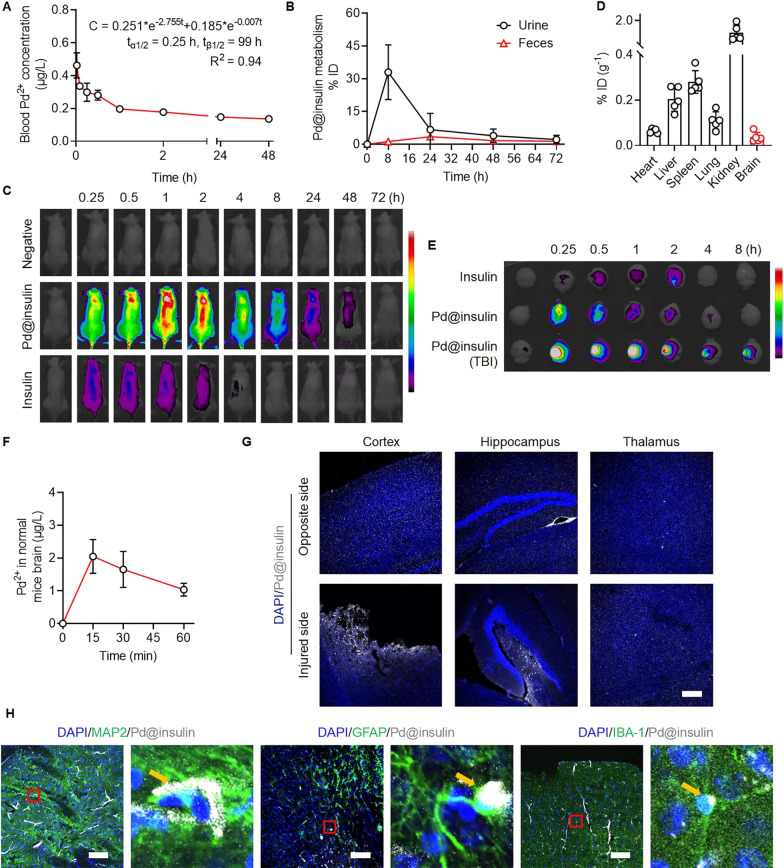
Pd@insulin clusters cross the BBB and aggregate in the injured cortex after TBI. **A** In vivo blood pharmacokinetic curve. **B** Excretive Pd^2+^ concentration in mice urine and feces (n = 3). **C** In vivo fluorescence images of control mouse (left), and mouse treated with Pd@insulin (middle) or insulin (right), respectively. **D** Biodistribution of Pd@insulin in major tissues of mice at 15 min post treatment. **E** Fluorescence images of brain tissue post insulin or Pd@insulin administration. **F** Pd^2+^ concentration in normal mice brain (after perfusion). Immunofluorescent staining of TBI mice for **G** different brain area including cortex, hippocampus and thalamus. **H** Immunofluorescent staining of TBI mice cortex (MAP2, Iba1 and GFAP on behalf of neuron, microglia and astrocyte, respectively) (Scale bar 100 μm). Data are all shown as mean ± SD.

The ability of intravenously injected Pd@insulin clusters to circulate through the blood in mice was assessed by a two-compartment pharmacokinetics model, and the half-lives of the central and peripheral components were 0.25 and 99 h, respectively (Fig. 4A). Pd@insulin clusters were excreted via the urine and feces (Fig. 4B, Additional file 1: Figs. S17, S18), and the peak excretion of Pd^2+^ in the urine and feces occurred 8 and 24 h, respectively, after intravenous injection, indicating the rapid metabolism of the Pd@insulin clusters.

To further analyze the biodistribution of Pd@insulin, Pd@insulin was labelled with Cy5 for *in vivo* fluorescence imaging (Fig. 4C). After intravenous administration, Pd@insulin was rapidly distributed throughout the whole bodies of mice with a metabolism time of 48 h, which is significantly longer than that of insulin (4 h). After PBS perfusion, Pd@insulin mainly enriched in kidney, spleen, and liver (Fig. 4D); the accumulation of Pd@insulin in the liver and kidney implied that Pd@insulin can be metabolized through the urine and feces (Fig. 4B). Kidney-urine and liver-feces are two main metabolic pathways for nanoparticles, and the sizes of nanoparticles determine the destiny. Previous studies found that the nanoparticles with sizes less than 5.5 nm could be effectively cleared from the blood to the renal tubules through the glomerular basement membrane [19, 25], matching with our observations.

Afterwards, we validated the BBB-penetrating ability of Pd@insulin. The time-dependent enrichment of Pd@insulin-Cy5, as measured by fluorescence imaging, suggested that Pd@insulin accumulated in the brain (Fig. 4E). This result was corroborated by examining the variation in Pd^2+^ concentration in the brain (Fig. 4F). Although the BBB is disrupted after TBI, it is still important for drugs to be able to cross the BBB due to the limited time window of spontaneous BBB recovery (Additional file 1: Fig. S19) [26]. Our observation proved the feasibility of delivering Pd@insulin clusters into the brain. We utilized insulin to allow the nanomaterial to cross the BBB because insulin can carry nanodrugs across the BBB via insulin-mediated transcytosis [17]. Pd@insulin clusters are much smaller (ca. 3.2 nm) and can better accumulate in the brain [13], than other insulin-based nanoparticles that are able to cross the BBB, such as insulin-coated HSA nanoparticles (ca. 150 nm), gold nanoparticles (ca. 20 nm) and insulin-conjugated nanogel (ca. 93 nm) [17] (Fig. 2B). The blood-delivered nanoparticles with smaller size are more likely to cross blood-brain-barrier [27–29], presumably due to the increase of paracellular permeability or the promotion of the internalization of the endothelial cells induced by nanoparticles [30]. Thus, Pd@insulin clusters may have important benefits, such as the ability to be rapidly metabolized and better therapeutic effects.

The accumulation of Pd@insulin at the site of TBI was also investigated. Pd@insulin was highly enriched at the injury site of TBI at all tested time points from 15 min to 8 h (Fig. 4E). In brain slices from TBI mice, Pd@insulin clusters showed greater aggregation in the injured cortex and hippocampus (Fig. 4G). Pd@insulin clusters were also detected in MAP2^+^ neurons, Iba1^+^ microglia, and GFAP^+^ astrocytes at the injury site, suggesting the uptake of Pd@insulin by brain cells (Fig. 4 H). In addition, TEM images with EDX analysis proved the presence of Pd in the cortex, kidney and intestine, verifying the above metabolic results (Additional file 1: Fig. S20). Therefore, we proved that Pd@insulin can across the BBB and accumulated at the injury site of TBI in mice, causing it to have better ROS scavenging through a durable cyclic catalytic reaction. The accumulation of Pd@insulin at the injured area is presumably due to the disturbed BBB integrity that leads to the blood exudate with the Pd@insulin nanoclusters, and the aggregation of microglia with great phagocytic capacity of Pd@insulin in the injured site.

### Pd@insulin clusters reverse excessive ROS accumulation of TBI after treatment

**Fig. 5:**
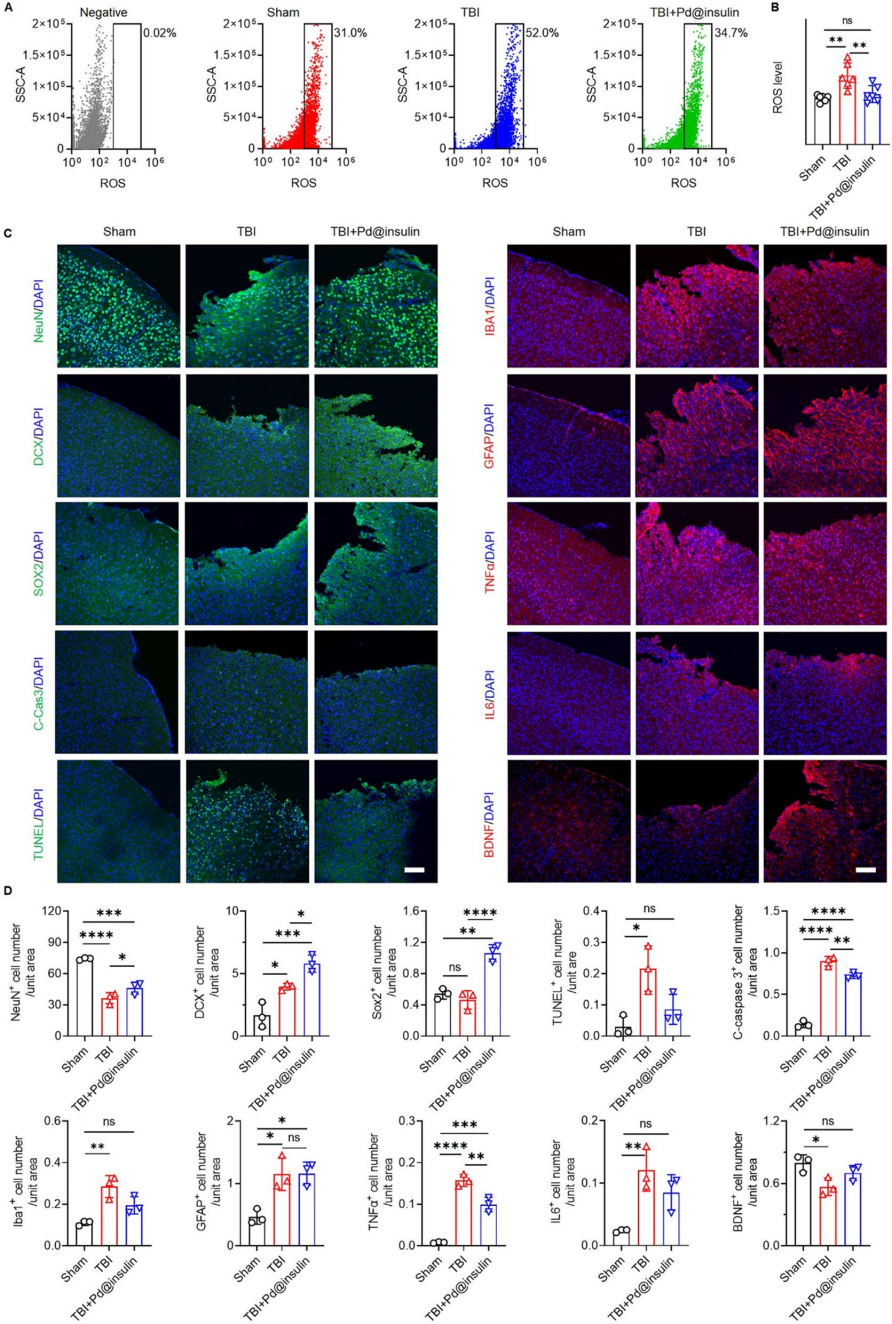
Pd@insulin clusters reverse excessive ROS accumulation, rescue neuronal loss, inhibit microglial activation, and reduce apoptosis after TBI. **A** Brain ROS level by flow cytometry analyses for Negative, Sham, TBI and TBI mice treated by Pd@insulin mice (10,000 cells per group), and **B** quantitative analysis (n = 6). **C** Representative immunofluorescent staining images of NeuN, doublecortin, Sox2, cleaved caspase 3, IBA1, GFAP, TNFα, IL6, BDNF, and TUNEL in injured cortex of TBI mice (Scale bar 100 μm), and **D** corresponded quantitative analyses (n = 3). Data are all shown as mean ± SD. Statistical analysis of (**B**,** D**) was performed by one-way ANOVA with a Tukey post hoc test.

We next measured ROS levels in the brains of TBI mice using flow cytometry analysis and a DCF ROS Assay Kit. ROS levels were significantly increased after TBI, as indicated by a significant increase in the number of ROS^+^ cells in the brains of TBI mice compared with those of control mice or sham mice (Additional file 1: Fig. S21A–E). Following TBI, mice were intravenously injected with Pd@insulin for 6 successive days, and ROS levels in the mouse brain were measured on the 7th day. The results revealed that Pd@insulin administration reduced the proportion of ROS^+^ cells in the brains of TBI mice from 52 to 34.7%, which was comparable to that in the brains of sham mice (31%) (Fig. 5A). A fluorescent quantitative ROS assay kit confirmed that TBI induced ROS accumulation in the mouse brain and that this accumulation was abrogated by Pd@insulin treatment (Fig. 5B), indicating the effects of Pd@insulin in scavenging excess ROS in the brains of TBI mice.

### Pd@insulin clusters rescue neuronal loss, inhibit microglial activation, and reduce apoptosis

The therapeutic effects of Pd@insulin on TBI were determined. Pd@insulin treatment resulted in a reduction in the size of the injury area after TBI (Additional file 1: Fig. S22). Immunohistochemical analysis demonstrated a significant increase in the proportions of mature NeuN^+^ neurons, DCX^+^ neural precursors, and Sox2^+^ neural stem cells (NSCs) in the injured cortex in Pd@insulin-treated TBI mice (TBI + Pd@insulin) compared with untreated TBI mice (Fig. 5C, D), indicating that Pd@insulin promoted neurogenesis and expansion of the NSC pool [31]. Subsequently, the proportion of activated microglia (Iba1^+^) was significantly reduced in the brains of Pd@insulin-treated mice compared with those of control TBI mice, suggesting that Pd@insulin treatment restrained TBI-induced microglial activation (Fig. 5C, D). Pd@insulin injection also reduced and increased the proportions of cells immunoreactive for proinflammatory cytokines (TNFα and IL6) and brain-derived neurotrophic factor (BDNF), respectively, in the damaged cortex, confirming that Pd@insulin exerted anti-inflammatory effects after TBI (Fig. 5C, D). Furthermore, Pd@insulin administration abolished TBI-induced apoptosis in the injured cortex, as indicated by reductions in the proportions of cleaved-caspase3^+^ and TUNEL^+^ cells in the brains of Pd@insulin-treated TBI mice (Fig. 5C, D). Therefore, we demonstrated that Pd@insulin-mediated ROS scavenging in the injured cortex enhanced neuroregeneration, inhibited neuronal loss, restrained neuroinflammation, and suppressed apoptosis.

### Motor function, cognitive and spatial memory amelioration

**Fig. 6:**
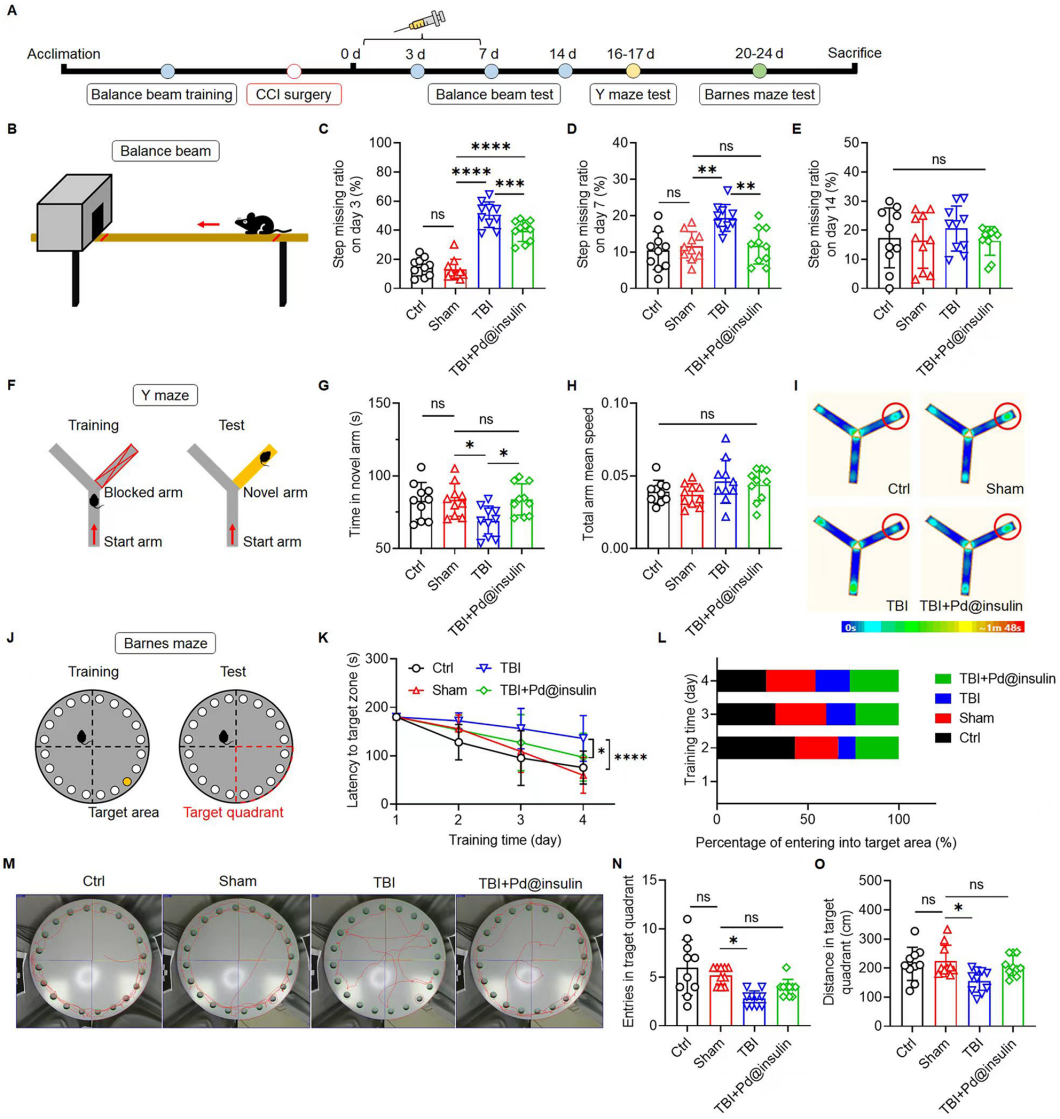
Pd@insulin clusters ameliorate motor function, cognitive and spatial memory impairment in TBI mice. **A** Schematic illustration of TBI, treatment and behavior tests. **B** Balance beam test: step missing ratio on **C** day 3 **D** day 7 and **E** day 14 (n = 10). **F** Y maze test: **G** time in novel arm **H** mean speed and **I** representative heat map of mice track (n = 10). **J** Barnes maze test: **K** latency to target area **L** percentage of mice that successfully find and enter into target area **M** representative track (red line) and **N** entry times in target quadrant **O** moving distance in target quadrant on test day (n = 10). Data are all shown as mean ± SD. Statistical analysis of (**C**–**E**), (**G**, **H**), (**N**, **O**), and (**K**) was performed by one-way/two-way ANOVA with a Tukey post hoc test.

We further examined the functional recovery of Pd@insulin-injected TBI mice with the balance beam, Y maze, and Barnes maze tests (Fig. 6A). First, the balance beam test was employed to evaluate the motor ability of the mice, which was measured by recording the missed step ratio of the mice on a wooden beam (Fig. 6B). There was no significant difference in the missed step ratio between the control and sham mice on days 3, 7, or 14, but the missed step ratio of the TBI mice was significantly increased on day 3 and 7 (Fig. 6C–E). Mice treated with Pd@insulin showed fewer missed steps than TBI mice on days 3 and 7, suggesting that Pd@insulin treatment markedly accelerated the recovery of motor ability in TBI mice (Fig. 6C–E, Additional file 1: Video S1). There was no difference in the missed step ratio between the TBI + Pd@insulin and TBI groups on day 14, most likely due to the spontaneous recovery of the TBI mice.

Then, the cognition and memory of control and experimental mice were evaluated by the Y maze and Barnes maze tests (Fig. 6F, J). In the Y maze, Pd@insulin treatment significantly increased the exploration time of TBI mice in the novel arm; importantly, this tendency was not due to a difference in speed between the groups (Fig. 6G, H). The representative track heat maps and videos similarly revealed that Pd@insulin treatment improved the performance of TBI mice in the Y maze (Fig. 6I, Additional file 1: Video S2). The Barnes maze is a sophisticated behavioral test of mouse cognition and spatial memory (Fig. 6J). In the Barnes maze, TBI mice spent a longer time finding the target than control and sham mice from training day 2 to training day 4, and Pd@insulin treatment shortened the time TBI mice took to reach the target zone (Fig. 6K). In addition, Pd@insulin treatment enhanced the capacity of TBI mice to find the target area (Fig. 6L, M). On the testing day, TBI mice also displayed a significant reduction in the number of entries and distance travelled in the target quadrant, and Pd@insulin treatment improved the performance of TBI mice to a degree that these mice exhibited no significant differences compared with the sham group, indicating that Pd@insulin treatment alleviated the impairment of cognition and memory in TBI mice (Fig. 6N, O).

### **The therapeutic effect on TBI occurs*via*neuroinflammation inhibition**

**Fig. 7:**
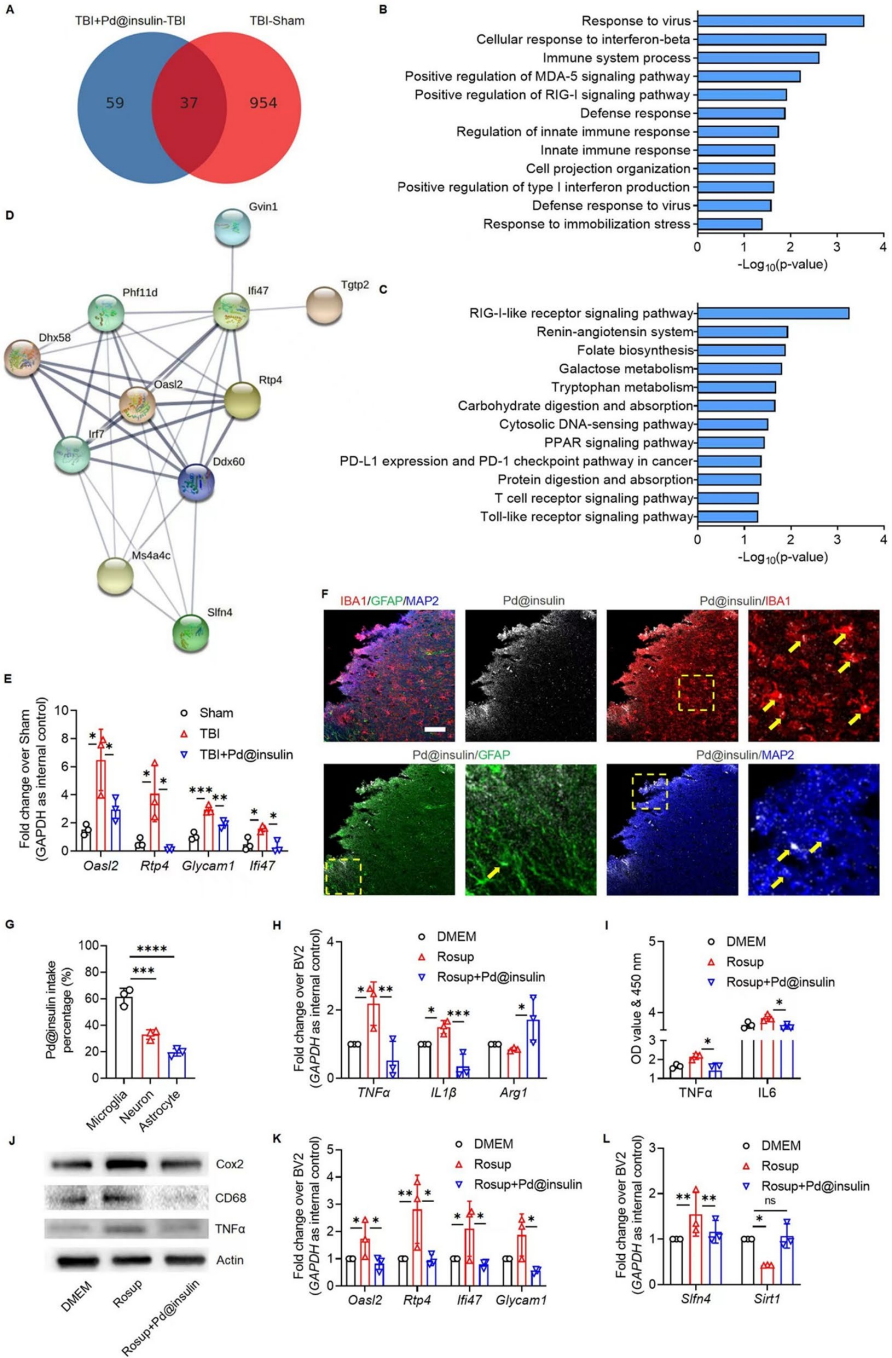
The therapeutic effect of Pd@insulin on TBI occurs via neuroinflammation inhibition. **A** Venn diagram of the transcriptomic profiles between TBI-Sham and Pd-TBI. **B** GO enrichment analysis and **C** KEGG pathway classification of 37 genes, screened out from the overlapped genes between TBI-Sham and TBI + Pd@insulin-TBI. **D** Protein-to-protein network of 37 genes involved in Pd@insulin treatment. **E** qRT-PCR analysis of mRNA expression of the genes (n = 3). **F** Representative immunofluorescent staining images of injured cortex in TBI mice post Cy5-label Pd@insulin injection (Scale bar 100 nm) and **G** quantitation analysis for the comparison of Pd@insulin intake in different brain cells (n = 3). qRT-PCR analysis of mRNA expression in BV2 treated by Rosup or Pd@insulin + Rosup (n = 3) for **H** M1/M2 related genes and **K** the genes from transcriptomics analysis or **L** related to neuroinflammation. The production of (**I** TNFα and IL6 in the supernatant of microglia determined by ELISA (n = 3). **J** Western blot of Rosup/Rosup + Pd@insulin treated microglia. Data are all shown as mean ± SD. Statistical analysis of (**E**, **G**, **H**, **I**, **K**, and **L**) was performed by one-way ANOVA with a Tukey post.

To further illuminate the mechanism underlying Pd@insulin-mediated recovery after TBI, transcriptome profiling of brain tissues (from the sham, TBI and TBI + Pd@insulin groups) was performed through RNA-seq. A total of 991 differentially expressed genes (DEGs), including 900 genes with upregulated expression and 91 genes with downregulated expression, were identified in the brains of TBI mice compared with those of sham mice (Additional file 1: Fig. S23A). In addition, 96 DEGs, including 60 genes with upregulated expression and 36 genes with downregulated expression, were identified in the brains of Pd@insulin-treated TBI mice compared with those of mice with TBI (Additional file 1: Fig. S23B). A Venn diagram of the two aforementioned groups of DEGs identified 37 overlapping genes related to the therapeutic effects of Pd@insulin (Fig. 7A). GO analysis suggested that the overlapping genes were enriched in GO terms associated with immune responses, such as response to virus, cellular response to interferon-beta, and immune system process (Fig. 7B). KEGG analysis indicated that these 37 genes were components of inflammation-related pathways, including RIG-I-like, T cell, and Toll-like signaling (Fig. 7C). Protein–protein interaction (PPI) network analysis identified an 11-gene network that is strongly associated with inflammatory responses, further indicating that Pd@insulin had marked effects on neuroinflammation (Fig. 7D). To validate our finding, we examined the expression of *Oasl2* [32], *Rtp4* [33], *Glycam1* [34], and *Ifi47* [35], 4 overlapping genes that are strongly associated with neuroinflammation, via qRT-PCR (Fig. 7E). The results revealed that Pd@insulin treatment significantly decreased the TBI-induced excessive expression of the aforementioned genes, confirming the RNA-seq results. Microglia-mediated neuroinflammation is one of the most pronounced reactions caused by TBI [36]. Microglia, which are resident immune cells of the CNS, are the first cells to respond to brain trauma, core neuroinflammatory cells, and the main phagocytes in the brain. Once activated, microglia release excessive ROS, exacerbating TBI-induced damage [37]. Inhibition of microglial activation attenuates learning deficits and stimulates neurogenesis [38]. Moreover, microglial activation can persist for years in TBI patients and may contribute to neurodegeneration and brain function deficits [39]. Given the importance of neuroinflammation in TBI pathogenesis, our results implied that Pd@insulin might promote recovery after TBI by alleviating neuroinflammation.

Since neuroinflammation-related genes and pathways were the most altered genes and pathways in the brains of TBI mice after Pd@insulin treatment, we next clarified the effects of Pd@insulin on microglial activation. First, we determined whether Pd@insulin targeted microglia in the brain. Immunohistochemical analysis demonstrated that the proportion of Pd@insulin^+^ microglia was significantly higher than the proportions of astrocytes and neurons, suggesting that most Pd@insulin clusters were internalized by microglia (Fig. 7F, G). It is expected since Pd@insulin nanoclusters could be recognized as exogenous materials and be mainly internalized by microglia, the resident macrophages and the primary defenders in the brains, presumably via phagocytosis, a much more efficient cargo uptake mechanism than endocytosis that is utilized by astrocytes and neurons [40–42]. We then analyzed the inflammation-modulating effects of Pd@insulin in an *in vitro* model of oxidative stress-induced microglial activation established by treating BV2 cells with Rosup. Pd@insulin treatment abolished the elevation of the expression levels of proinflammatory (M1) microglia-specific genes (*TNF* and *Il1b*) and the reduction in the expression levels of anti-inflammatory (M2) microglia-specific genes (*Arg1*) (Fig. 7H). ELISA showed that Pd@insulin reversed the excessive release of the inflammatory cytokines TNFα and IL6 (Fig. 7I). The inhibitory effects of Pd@insulin on the expression of the inflammatory factors Cox2, CD68 and TNFα were assessed by Western blotting, which confirmed the anti-inflammatory effects of Pd@insulin (Fig. 7J). Furthermore, we found that Pd@insulin inhibited the transcription of *Oasl2*, *Irf7*, *Rtp4*, and *Ddx60*, which is consistent with the RNA-seq results (Fig. 7K). In addition, the abnormal expression of the oxidative stress-related genes *Slfn4* and *Sirt1* was reversed by Pd@insulin [43] (Fig. 7L). Therefore, our results revealed that Pd@insulin was mainly taken up by phagocytotic microglia and promoted recovery after TBI recovery, most likely by modulating the inflammatory responses of microglia.

## Discussion

The excessive production and accumulation of multiple ROS is a key pathological change in various acute brain damage and chronic neurodegenerative disorders [44]. The elimination of excessive ROS has been proved as a promising therapeutic strategy in treating neurological diseases [6]. Three main types of ROS scavengers have been generated including natural enzymes, antioxidant drugs, and nanozyme [6]. However, natural enzymes and antioxidant drugs are struggled with single ROS elimination ability, high cost, low stability, and recycle difficulties [45]. Nanozymes can overcome some aforementioned issues [46], however, their synthesis processes need to be simplified, their sizes require to be reduced, and their BBB crossing capacity demands to be improved. The rapid development of nanobiotechnology confers us a powerful tool to synthesize nanoparticles with bright prospects for clinical application [47, 48]. Here, we exploited ROS-scavenging Pd@insulin clusters for enhancing post brain injury recovery, prepared via a green and high-efficiency protein-incubated synthesis method. This method depends on protein-mediated biomimetic biomineralization and spatial confinement effect preventing particles aggregations. Compared with other chemical synthesis methods, such as precipitation, sol-gel, solvothermal synthesis, and hydrolyzation, the protein-incubated strategy is simply, green, and toxic reagents and harsh conditions (e.g., high temperature and high pressure) free [49]. Moreover, the obtained nanodrugs were ultrasmall (3.2 nm) and coated with natural proteins (Figs. 1 and 2), which further lessens toxicity and prolongs circulation time of nanoparticles in the blood [50]. Insulin was chosen as the reducer to mature Pd cluster and the stabilizer to avoid aggregation. Compared with other common protein templates such as BSA, ovalbumin, and ferritin [49], insulin possesses smaller molecular weight [51], positive therapeutic effect to TBI at low dose, and ability to across the BBB with nanoparticles [17]. Pd cluster was chosen to synthesize due to its specific electronic structure for ROS scavenging. Pd@insulin clusters exhibited great brain entering efficiency (Fig. 4) and outstanding ROS scavenging ability in solution, in vitro, and in vivo (Figs. 2 and 5 A). More importantly, Pd@insulin clusters aggregated at the injured site of TBI post intravenous administration, enhancing neuroprotection and neurogenesis, and suppressing neuroinflammation and apoptosis (Fig. 5B, C). Furthermore, Pd@insulin clusters were able to ameliorate the impaired motor function, cognition, and spatial memory of TBI mice (Fig. 6). Interestingly, most of clusters were phagocyted by microglia, shifting the pro-inflammatory phenotype to anti-inflammatory one (Fig. 7). Since the excessive ROS-driven pro-inflammatory microglial polarization is important for the pathogenesis of TBI [52], our study implied the inhibition of microglia-driven neuroinflammatory responses as the underlying mechanism of Pd@insulin-induced recovery after brain injury.

The toxicity of Pd@insulin clusters is key examined since Pd is a nonexistent element in mammal and insulin is a hypoglycemic drug [14]. Pd@insulin did not show cytotoxicity in all examined brain cell lines within test concentration (Additional file 1: Fig. S11). Pd@insulin could be rapidly excreted by kidney-urine and liver-feces pathways (8 h) after intravenous injection due to the low molecular weight of insulin and the ultrasmall size (3.2 nm) of Pd@insulin clusters. Moreover, although Pd@insulin clusters reduced the blood glucose in once administration, the daily blood glucose remained normal after continuous treatment, leading to no death of Pd@insulin-injected mouse (Fig. 3). Hence, the safety of Pd@insulin clusters has been proved via both *in vitro* and *in vivo* evaluations. Interestingly, unlike Pd@insulin, insulin exhibited significant cytotoxicity (Additional file 1: Fig. S11) and caused animal death due to the over depressed blood glucose (Fig. 3). These differences between insulin and Pd@insulin may be attributed to the insulin change via chemical synthesis, because the alkali synthesis environment induced parts of protein degeneration, and the existence of Pd cluster may impede insulin signal transduction [49].

## Conclusions

In summary, we generated novel Pd@insulin clusters via a highly efficient protein incubation synthesis method for the treatment of TBI. The ultrasmall clusters (3.2 nm) were able to cross the BBB and aggregate in the injured cortex and exhibited outstanding ROS-scavenging ability while lessening the side effects of insulin. The administration of Pd@insulin resulted in substantial behavioral recovery in TBI mice and ameliorated pathological changes. The Pd@insulin clusters most likely exerted their therapeutic effects by attenuating oxidative stress-induced microglial activation, resulting in a reduction in inflammatory responses and neuronal loss after TBI. Hence, our study demonstrated that Pd@insulin clusters are promising nanodrugs for the treatment of TBI and other ROS-related neurological disorders.

## Materials and methods

### Reagents

Sodium tetrachloropalladate (II) (Na_2_PdCl_4_, > 98%) was purchased from Sigma-Aldrich Trading Co., Ltd. (Shanghai, China). Insulin (bovine pancreatic, 27 u/mg), sodium hydroxide (NaOH), hydrochloric acid (HCl, 36–38%) and nitric acid (HNO_3_, 65–68%) were obtained from Sinopharm Chemical Reagent Co., Ltd. (Shanghai, China). A superoxide anion assay kit, hydroxyl radical assay kit and H_2_O_2_ detection kit were purchased from Nanjing Jiancheng Bioengineering Institute (Nanjing, China). A Cell Counting Kit-8 (CCK-8) assay kit was obtained from Beyotime Institute of Biotechnology (Haimen, Jiangsu, China), and an ROS assay kit was purchased from Abcam Inc. (ab238535). PBS was obtained from Procell Life Science and Technology Co., Ltd. (Wuhan, China). Ultrapure water (18.2 MΩ cm^− 1^) was purified with a Milli-Q system.

### Synthesis of Pd@insulin clusters

Pd@insulin clusters were prepared by a biomimetic insulin incubation method. Insulin powder (30 mg) was routinely dissolved in 50 mL deionized water at 37 °C with magnetic stirring, and 25 µL Na_2_PdCl_4_ solution (100 mM) was added gradually. Subsequently, the pH of the solution was adjusted to 10–11 with 1 M NaOH. After 24 h, a pellucid Pd@insulin cluster solution was obtained and dialyzed (Mw = 3 kDa) against ultrapure water. A Pd@insulin cluster powder was obtained by freeze drying.

### Characterization of Pd@insulin clusters

TEM and high-resolution transmission electron microscopy (HRTEM) were conducted with a JEM-2100 microscope operated at 200 kV and a JEM-2100 microscope equipped with an EDX energy-dispersive spectrometer. SEM was performed with a Hitachi S4800 microscope operated at 3 kV. XPS was conducted on a PHI-5000 C ESCA system (Perkin Elmer) with Mg Kα radiation using an internal standard (C1s peak at 284.6 eV). CD was measured by a Thermo Scientific Nicolet iS10 spectrometer in the range of 4000–400 cm^− 1^ and a Jasco J-815 spectrophotometer. Size and stabilization were evaluated by dynamic light scattering (DLS, Nano-ZS90, Malvern) and ultraviolet-visible (UV-vis) spectrophotometry (Nanodrop 2000, Thermo Fisher). TGA was conducted by Mettler Toledo TGA/DSC3+. The ion concentration was measured by inductively coupled plasma mass spectrometry (ICP-MS, iCAP RQ, Thermo Fisher).

### ROS-scavenging ability of Pd@insulin clusters in solution

The H_2_O_2_, O_2_*^−^ and OH* scavenging capacities of the clusters were determined by assay kits. H_2_O_2_-scavenging capacity was evaluated by reacting H_2_O_2_ with ammonium molybdate, which resulted in the formation a yellow solution with an absorbance peak at 405 nm. The concentrations of superoxide anion, which was generated by the reaction between xanthine and xanthine oxidase, and hydroxyl radical, which was produced by the Fenton reaction, were determined at a wavelength of 550 nm via chromogenic Griess reagent. EPR was performed to determine the free radical (superoxide anion and hydroxyl radical)-scavenging ability of the Pd@insulin clusters. hydroxyl radical, which was generated from H_2_O_2_ by UV-laser irradiation, was captured by BMPO, while superoxide anion, which was generated by the reaction between 2.5 mM KO_2_ and 3.5 mM 18-crown-6, was captured by DMPO. The magnetic field signals of hydroxyl radical and superoxide anion were detected by spectrometry (Bruker A300, Germany).

### Density functional theory (DFT) calculation

The palladium cell was cleaved (220) crystal plane to build (3 * 3 * 1) supercell, and the Pd (220) supercell of five original layers were fixed in the next two layers and relaxed the rest of the atomic layers. All the self-consistent periodic DFT calculations were carried out using the DMol3 code as implemented in the Materials Studio package. The electron exchange and correlation were described with GGA-PBE functional, while the DFT-D (G06) method was used in DFT calculations for the dispersion correction. The localized double-numerical quality basis set with a polarization d-function (DNP-3.5 file) was chosen to expand the wave functions. The core electrons of the metal atoms were treated using the effective core potentials (ECP), and the orbital cutoff were 4.0 Å for all atoms. Brillouin-zone integrations were performed on a k-point mesh sampling grid of 3*2*1 by Monkhorst-Pack with a thermal smearing of 0.008 Ha. For the geometry optimization, the convergences of the energy, Max. force, and Ma. displacement was set as 1 × 10 ^− 5^ Ha, 2 * 10 ^− 3^ Ha/Å, and 5 * 10 ^− 3^ Å, and the SCF convergence for each electronic energy was set as 1.0 * 10^–6^ Ha. All the transition states (TSs) of the elementary reactions are identified using a complete linear synchronous transit and quadratic synchronous transit (LST/QST) approach. The convergence criterion was set for the root-mean-square forces on the atoms to be 0.01 Ha/Å.

### Cell culture

Three cell lines, i.e., N2a, BV2 and A172 cells, were used. N2a and BV2 cells were cultured in Dulbecco’s modified Eagle’s medium (DMEM) supplemented with 10% fetal bovine serum (FBS) with 1% streptomycin and penicillin in a 37 °C incubator, whereas A172 cells were cultured in DMEM/F12 containing the same concentrations of FBS and antibiotics. Primary microglia were cultured in DMEM containing 10 ng/mL GM-CSF, 10% FBS, 1% streptomycin and penicillin.

### In vitro cluster uptake

N2a, BV2 and A172 cells were seeded into 24-well plates. After 24 h, Cy3 (Cy3-NHS, GLPBIO)-labelled Pd@insulin (Pd@insulin-Cy3) was added to the cells for 1, 2, 4, 6 or 8 h, and the cluster uptake efficiency of each cell type was assessed with a Zeiss AX10 fluorescence microscope and ZEN 2.3 (blue edition) software.

### ROS-scavenging ability of Pd@insulin clusters in vitro

A commercial ROS assay kit consisting of a DCFH-DA probe and Rosup (a positive control) was employed to evaluate the *in vitro* ROS-scavenging capacity of Pd@insulin. N2a, BV2 and A172 cells were seeded in 24-well plates, and high ROS levels were induced by Rosup (50 µg/mL, 20 min) treatment. Then, the medium was replaced with Pd@insulin or insulin solution for 2 h. Afterwards, the cells were washed with serum-free medium three times to remove the free clusters/insulin, and fresh medium containing 10 µM DCFH-DA probe was added for 30 min. Finally, the cells were washed three times with serum-free medium for fluorescence imaging.

### Evaluation of the biocompatibility of Pd@insulin clusters in vitro

The cytotoxicity of the clusters was determined by the CCK-8 assay. N2a, BV2 and A172 cells were seeded in 96-well plates at a density of 10^4^ cells per well and incubated at 37 °C and 5% CO_2_. After 24 h of incubation, the medium was removed and replaced with fresh culture medium containing different concentrations of Pd@insulin/insulin (Additional file 1: Table S2). After 1 or 2 days of incubation, the culture medium was replaced with CCK-8 solution (10 µL per 100 µL medium), and the cells were incubated for 30 min. Then, cell viability was quantified by measuring the absorbance at 450 nm with a microplate reader (Versa Max, Molecular Devices).

### Mice

C57BL/6J mice were housed and bred at the Comparative Medicine Animal Facilities of Tongji University School of Medicine. All procedures were conducted according to protocols approved by the Institutional Animal Care and Use Committee of Tongji University School of Medicine (reference number SYXK (HU) 2014-0026).

### Analysis of blood glucose levels in mice

Fresh blood (5 µL) was obtained from the tail of each mouse, and blood glucose levels were measured with a commercial glucometer (Yuyue 580, Jiangsu).

### Evaluation of the biocompatibility of Pd@insulin clusters in vivo

Eight- to ten-week-old male mice were treated with 250 µL Pd@insulin for 6 successive days (intravenously). ECG was conducted to evaluate mouse heart function (Labchart, ADInstruments). Major tissues, including heart, liver, spleen, lung, kidney and brain tissues, were isolated for H&E staining, and blood samples were collected for blood panel analysis and serum biochemistry tests, i.e., analysis of alanine aminotransferase (ALT), aspartate aminotransferase (AST), ALB, TBil, urea and Cre levels. The presence of occult blood and transferrin in excreted faeces was assessed with test strips.

Depression- and anxiety-like behaviors were assessed by the TST, FST, and SPT. Eight- to ten-week-old male mice were treated with 250 µL Pd@insulin for 6 successive days (intravenously). On day 7, each mouse was suspended by the tail with its head 25 cm away from a table using adhesive tape for 6 min, and the immobility time in the final 4 min was recorded with a camera. In the FST, the mice were placed in a glass cylinder (height: 30 cm, diameter: 20 cm) filled with water (23–25 °C) to a depth of 15 cm for 6 min, and immobility time in the final 4 min was recorded. To explore the sucrose preference of cluster-treated mice, all mice were exposed to two bottles of water, two bottles of 2% sucrose solution, and water deprivation for 24 h in advance. Finally, each mouse was given one bottle of water and one other bottle of 2% sucrose solution, and sucrose preference was calculated by determining the ratio of water consumed to sucrose solution consumed.

### BBB-crossing ability of Pd@insulin clusters

To explore the ability of the clusters to cross the BBB, Cy5-NHS (APExBIO) was used to label Pd@insulin (Pd@insulin-Cy5) and insulin (insulin-Cy5). Pd@insulin clusters (1 mL) were incubated with Cy5-NHS (1 mg/mL) for 1 h at room temperature. Extra free dyes were removed by repetitive ultrafiltration centrifugations to obtain labeled Pd@insulin clusters. After intravenous injection, the distribution of the labeled Pd@insulin clusters throughout the bodies of the mice was recorded at different time points by an in vivo fluorescence imaging system (VISQUE In vivo Smart-LF, Vieworks), and the distribution in brain tissue was assessed. To further prove that the clusters were able to cross the BBB, the Pd^2+^ ion concentration was measured by ICP-MS, and brain sections from cluster-treated mice were subjected to TEM and EDX analysis and immunohistochemical staining (DAPI, 1:1000; MAP2, mouse, cat# MB0078, Bioworld, 1:100; Iba1, goat, cat# ab5076, Abcam, 1:100; GFAP, chicken, cat# AB5541, Millipore, 1:500).

### Pharmacokinetics and biodistribution of Pd@insulin clusters

The pharmacokinetics of Pd@insulin was assessed by measuring the Pd^2+^ ion concentration in mouse blood and analyzed by a two-compartment pharmacokinetic model. At different time points (1 min, 5 min, 15 min, 30 min, 1 h, 2 h, 24, and 48 h), fresh blood (5 µL) was obtained from the tails of Pd@insulin-treated mice and dissolved in aqua regia for quantification by ICP-MS. The biodistribution of the clusters was quantified by determining the Pd^2+^ concentration in tissue. To avoid interference by blood, Pd@insulin-treated mice were perfused with PBS, and major tissues, including heart, liver, spleen, lung, kidney and brain tissues, were isolated. To assess metabolism, the feces and urine of cluster-treated mice were collected at different time points (8 h, 24 h, 48 and 72 h). Finally, major tissues, feces and urine were dissolved in aqua regia for 2 days and passed through a 0.22 μm filter. The Pd^2+^ concentration in the obtained solution was determined by ICP-MS. Kidney and intestine sections were observed by TEM and EDX analysis.

### TBI model

Severe controlled cortical impact (CCI) injury was induced in 8- to 10-week-old male mice. The mice were routinely anaesthetized with 4% chloral hydrate and placed in a stereotaxic frame (Kopf Instruments, Tujunga, CA). After sterilization, the scalp was retracted, and a 4 mm craniotomy centered between the lambda and bregma sutures was performed. The skull pieces were carefully discarded, taking care to prevent disruption of the underlying dura disruption. Subsequently, the angle between the impacting piston and exposed cortex was corrected, and the mice underwent impact (deformation: 1.2 mm; piston velocity: 3.05 m/sec) (Impact One TM Stereotaxic Impactor for CCI, Leica Microsystem). Sham mice underwent anesthesia but did not undergo CCI.

### Treatment of TBI mice

The mice were randomly divided into the naïve (control), sham and TBI by CCI groups. After surgery, the TBI mice were randomly divided into two groups: a group intravenously injected with 250 µL Pd@insulin cluster solution for 6 days and a group treated with the same volume of PBS.

### ROS-scavenging ability of Pd@insulin clusters in TBI mice

Brain tissues were isolated from euthanized mice, minced for 30 min, and digested with 2.5% trypsin, and a single-cell suspension was obtained by passing the tissues through filters twice (70 μm and 40 μm). According to the protocol of the assay kit, catalyst and DCFH solution were successively mixed with the single-cell solution. After 30 min and three rinses in serum-free medium, the fluorescent ROS signal was analyzed by flow cytometry (BD-LSRFortessa, BD).

### Behavior tests

*Balance beam test* The motor function of the mice was evaluated by the balance beam test. The mice were subjected to training 3 days before CCI, and the test was performed on days 3, 7 and 14 after TBI. Each mouse was placed on the starting point on a wooden beam (width: 0.5 cm, length: 100 cm) elevated 60 cm above the ground. On the training days, the mice were trained to cross the beam and enter a closed black box spontaneously. If a mouse failed to cross the beam, it was gently guided forward. On the testing days, the average time to cross the beam, total step number, and missed step ratio were recorded manually.

*Y maze test* The Y maze consisted of 3 grey glass arms (length: 30.5 cm, width: 9 cm, height: 14.5 cm) at a 120° angle to each other. The mice were habituated to two arms for 6 min, during which the other arm was blocked. On the testing day, the blocked arm (defined as the novel arm) was opened, and the time spent the novel arm (in the final 4 min of a 6-min period) was recorded for each mouse with a video camera.

*Barnes maze test* A circular acrylic plastic table (diameter: 115 cm) with 18 holes (diameter: 7 cm) spaced equidistant around the perimeter was used. Training was performed for 4 days, during which each mouse was placed in the center of the maze and covered with an opaque bowl for 10 s before being allowed to explore the maze and locate and enter an escape box beneath the table in the presence of noise interference. The latency to enter the target zone was recorded. If a mouse failed to find the escape box within 5 min, it was gently guided to enter the target. On the testing day, the escape box was replaced, and the tendency of the mice to explore the primary escape box was evaluated by measuring the number of entries into the target quadrant and distance travelled in the target quadrant.

### Immunofluorescence staining

Brain sections were fixed in 4% paraformaldehyde, washed three times with PBS, and incubated in permeabilization and blocking buffer (3% BSA, 10% donkey serum, and 1% Triton X-100 in PBS) for 1 h. The brain sections were incubated with primary antibodies including GFAP (chicken, cat# AB5541, Milipore, 1:500), Iba1 (goat, cat# ab5076, Abcam, 1:100), NeuN (mouse, cat# MAB377, Sigma-Aldrich, 1:500), DCX (rabbit, cat# 4604, Cell Signaling Technology, 1:800), Sox2 (mouse, cat# 4900s, Cell Signaling Technology, 1:200), TNFα (rabbit, cat# ab183218, Abcam, 1:500), IL6 (rabbit, cat# 66146-1, Proteintech, 1:50), BDNF (rabbit, cat# ab108319, Abcam, 1:500), and cleaved-caspase3 (rabbit, Cell Signaling Technology, cat# 9664s, 1:400) overnight at 4 °C. Then, all sections were washed with PBS and incubated with secondary antibody *for 1 h. Immunofluorescence was observed under a confocal microscopy (FV3000, Olympus).*

### TUNEL

Mouse brain sections were washed twice with PBS and treated with a mixture of labelling solution and enzyme solution (In Situ Cell Death Detection Kit, TMR red, Sigma) for 1 h at 37 °C; sections not treated with enzyme solution were used as negative controls. Then, the sections were washed twice with PBS and observed by confocal microscopy.

### Transcriptome analysis

Mice were randomly divided into the sham, TBI and TBI + Pd@insulin groups and sacrificed for brain tissue collection after 6 days of treatment. Total RNA was extracted using a mirVana miRNA Isolation Kit (Ambion) following the manufacturer’s protocol. RNA integrity was evaluated using an Agilent 2100 Bioanalyzer (Agilent Technologies, Santa Clara, CA, USA), and samples with an RNA integrity number (RIN) ≥ 7 were used for subsequent analysis. Libraries were constructed using a TruSeq Stranded mRNA LTSample Prep Kit (Illumina, San Diego, CA, USA) according to the manufacturer’s instructions. These libraries were sequenced on the Illumina sequencing platform (HiSeqTM 2500 or Illumina HiSeq X Ten), and 125-bp/150-bp paired-end reads were generated.

Transcriptome sequencing and analysis were conducted by OE Biotech Co Ltd. (Shanghai, China). Raw data (raw reads) were processed using Trimmomatic. Reads containing poly N and low-quality reads were removed to obtain clean reads. Then, the clean reads were mapped to the reference genome using HISAT.

The FPKM and read counts value of each transcript (protein-coding) were calculated using bowtie2 and eXpress. DEGs were identified using the DESeq (2012) functions estimateSizeFactors and nbinomTest. A P value < 0.05 and a fold change > 1.5 or fold change < 0.5 were set as the thresholds for significantly different expression. Hierarchical cluster analysis of DEGs was performed to assess transcript expression patterns. GO enrichment analysis and KEGG pathway analysis of the DEGs were performed using R based on hypergeometric distribution.

PPIs were analyzed by the Search Tool for the Retrieval of Interacting Genes/Proteins (STRING) algorithm.

### Quantitative real-time polymerase chain reaction (qRT-PCR)

cDNA was generated from mRNA using OligodT primers with a Transcriptor First Strand cDNA Synthesis Kit (HiScript III All-in-one RT SuperMix Perfect for qRT-PCR, Vazyme). An RNase inhibitor was used to prevent degradation. Amplification was performed using Taq Pro Universal SYBR qRT-PCR Master Mix (Vazyme) and specific primer sets (Supplementary Table 3). mRNA expression levels were normalized by to the expression of *GAPDH*.

### ELISA

The concentrations of TNF-α (abs520010, ABSIN) and IL-6 (KMC0021, Invitrogen) in the microglial culture medium were measured following the manufacturer’s protocol.

### Western blot

Protein was extracted from microglia using M-PER Protein Extraction Buffer (Pierce) containing protease inhibitor cocktail (Sigma). The protein concentration was determined via the BCA method. The proteins were separated by sodium dodecyl sulfate polyacrylamide gel electrophoresis (SDS-PAGE) and electrophoretically transferred on to polyvinylidene fluoride membranes (Millipore and Bio-Rad). The membranes were incubated with primary antibodies against COX2 (rabbit, cat# ab15191, Abcam, 1:1000), CD68 (rabbit, cat# ab125212, Abcam, 1:1000), β-actin (mouse, cat# a5441, Sigma, 1:5000), and TNFα (rabbit, cat# ab183218, Abcam, 1:1000) overnight and then incubated with a horseradish peroxidase-linked anti-rabbit or anti-mouse secondary antibody (Cell Signaling Technologies, 1:5000). The bands were visualized with Pierce ECL Western Blotting Substrate (Thermo Fisher Scientific, Waltham, MA, United States).

### Statistical analyses

Differences between two independent groups were analyzed by unpaired Student’s* t*-test, and differences between multiple groups were analyzed by one-way/two-way ANOVA followed by Tukey’s post hoc test. The data are shown as the mean ± SD, and P < 0.05 was considered significant.

## Supplementary Information

**Additional file 1: Figure S1.** TEM images of Pd@insulin synthesized by different ratio of protein to Pd^2+^ concentration (**A**) insufficiency (**B**) proper and (**C**) excessive amounts of insulin. The concrete ratio was listed in Supplementary table 1. (Scale bar 100 nm). **Figure S2.** The solution variation from insulin to Pd@insulin clusters via the protein-incubated method. **Figure S3.** (**A**) XPS analysis of the Pd peak of Pd@insulin clusters. (**B**) CD spectrum (arrows point out the peaks of α-helix) and (**C**) Thermo gravimetric analyses (TGA) of Pd@insulin clusters and insulin. **Figure S4.** SEM image of Pd@insulin powder, with element mapping and EDX analyses. **Figure S5.** (**A**) DLS and (**B**) UV–vis analyses of Pd@insulin before and after lyophilization. **Figure S6.** DLS size variation of Pd@insulin re-dissolved in double distilled water (ddWater), PBS, DMEM and saline (n = 3). **Figure S7.** UV–vis spectrum of Pd@insulin re-dissolved in (**A**) ddWater (**B**) PBS (**C**) DMEM and (**D**) saline. **Figure S8.** The fate of H_2_O_2_ evaluation after Pd@insulin nanoclusters treatment with different times via Griess reagent (Strong absorbance at 550 nm indicate the free radicals’ formation). Data are all shown as mean ± SD. Statistical analysis of was performed by one-way ANOVA with a Tukey post hoc test. **Figure S9.** Multiple rounds ROS scavenging evaluation for Pd@insulin (result indicates no performance loss of Pd@insulin nanocluster in ROS scavenging reaction). Data are all shown as mean ± SD. Statistical analysis of was performed by unpaired Student’s t-test. **Figure S10.** N2a, BV2 and A172 cell lines intake efficiency of Cy3-labeled Pd@insulin at different time point (Scale bar 200 μm). **Figure S11.** In vitro cytotoxicity of Pd@insulin clusters towards N2a, BV2, and A172 cells for (**A**) 24 h and (**B**) 48 h (n = 5, * represents the significant difference between Ctrl, # represents the significant difference between Pd@insulin and insulin) (n = 5). Data are all shown as mean ± SD. Statistical analysis of was performed by one-way ANOVA with a Tukey post hoc test. **Figure S12.** Hemolysis evaluation of Pd@insulin with different concentration (n = 5). Data are all shown as mean ± SD. **Figure S13.** The ability of Pd@insulin (blue arrowhead) to reduce blood glucose equivalently to 14% pure insulin solution (n = 3), the blood glucose detect was post Pd@insulin treatment for 1 h. Data are all shown as mean ± SD. **Figure S14.** Serum biochemistry between normal mice (Ctrl) and Pd@insulin-treated mice (n = 3). Data are all shown as mean ± SD. Statistical analysis was performed by unpaired Student’s t-test. **Figure S15.** Representative H&E staining of mice main tissues post Pd@insulin intravenous injection for successive 6 days (Scale bar 1 mm). **Figure S16.** Feces examination from normal mice (Ctrl) and Pd@insulin-treated mice (1 indicates occult blood positive, 2 indicates transferrin positive, and C indicates both negative) (n = 5). **Figure S17.** (**A**) TEM images of mice urine at different time points post Pd@insulin intravenous injection (Scale bar 100 nm) and (**B**) Pd weight ratio by EDX analysis. Data are all shown as mean ± SD. **Figure S18.** SEM image with mapping and EDX analysis of the fracture surface of mice feces at 24 h post Pd@insulin intravenous injection. **Figure S19.** Mice TBI processes. (**A**) Fixation and scalp open (**B**) and (**C**) craniotomy (**D**) and (**E**) impacting piston location (**F**), traumatic impact. **Figure S20.** TEM images of the cortex, kidney and intestine from normal and TBI mice, with EDX analysis, post Pd@insulin intravenous injection (Scale bar 1 μm). **Figure S21.** Total ROS level of mice brain. Flow cytometry analysis of ROS (10,000 cells per group) for (**A**) negative (**B**) naïve (Ctrl) (**C**) Sham and (**D**) TBI mice, and (**E**) quantitative analysis of ROS level from each mouse (n = 7–8). Data are all shown as mean ± SD. Statistical analysis was performed by one-way ANOVA with a Tukey post hoc test. **Figure S22.** Representative image of mice brains, from Ctrl, Sham, TBI, and TBI post successive 6-day Pd@insulin intravenous injections (The red circle showed injured cortex). **Figure S23.** Differential genes between (**A**) TBI and Sham (**B**) TBI and TBI post Pd@insulin treatment (TBI + Pd@insulin) (p < 0.05, fold change > 1.5, n = 5). **Table S1.** The exploration of the ratio of insulin to Pd^2+^ for Pd@insulin synthesis. **Table S2.** The drug concentration for treating cells. **Table S3.** Mice primers sequences for qPCR. **Video S1.** Balance beam test for mice motor ability test. **Video S2.** Y maze test for mice cognition and spatial memory test.

## Data Availability

All data needed to evaluate the conclusions in the paper are present in the paper and/or the Supplementary Materials. Additional data related to this paper may be requested from the authors.

## Abbreviations

ALB: Albumin
ALT: Alanine aminotransferase
Arg1: Arginase-1
AST: Aspartate aminotransferase
BBB: Blood-brain-barrier
BCA: Bicinchoninic acid
BDNF: Brain-derived neurotrophic factor
BSA: Bovine serum albumin
CAT: Catalase
C-Casp3: Cleaved caspase 3
CCI: Controlled cortical impact
CCK8: Cell counting kit-8
CD: Circular dichroism
Cre: Creatinine
DAPI: 4′,6-diamidino-2-phenylindole
DCX: Doublecortin
ddWater: Double distilled water
DFT: Density functional theory
DLS: Dynamic light scattering
DMEM: Dulbecco’s modified eagle medium
ECG: Electrocardiogram
EDX: Energy dispersive X-ray spectroscopy
ELISA: Enzyme-linked immunosorbent assay
EPR: Electron paramagnetic resonance
FBS: Fetal bovine serum
FST: Forced swimming test
FTIR: Fourier transform infrared spectroscopy
GFAP: Glial fibrillary acidic protein
GO: Gene ontology
H&E: Hematoxylin and eosin staining
HRTEM: High resolution transmission electron microscope
Iba1: Ionized calcium binding adapter molecule 1
ICP-MS: Inductively coupled plasma mass spectrometry
IL6: Interleukin-6
KEGG: Kyoto encyclopedia of genes and genomes
NeuN: Neuronal nuclei
MAP2: Microtubule associated protein 2
NSC: Neural stem cell
PBS: Phosphate buffered saline
qRT-PCR: Quantitative real-time polymerase chain reaction
ROS: Reactive oxygen species
SEM: Scanning electron microscopy
SOD: Superoxide dismutase
Sox2: SRY-box transcription factor 2
SPT: Sucrose preference test
TBI: Traumatic brain injury
TBil: Total bilirubin
TEM: Transmission electron microscope
TGA: Thermal gravimetric analysis
TNFα: Tumor necrosis factor-α
TST: Tail suspension test
TUNEL: TdT-mediated dUTP nick-end labeling
XPS: X-ray photoelectron spectroscopy
