# Supplementary material for: Insulin-incubated palladium clusters promote recovery after brain injury
Source: J Nanobiotechnology. 2022 Jun 25;20:299. doi: 10.1186/s12951-022-01495-6 (PMC9233827; doi:10.1186/s12951-022-01495-6)

**Supplementary Information for**

Insulin-Incubated Palladium Clusters Promote Recovery after Brain Injury

*Shengyang Fu^1ǂ^, Shu Zhao^1ǂ^, Huili Chen^1ǂ^, Weitao Yang^2,3,4^, Xiaohuan Xia^1,4,5,6*^, Xiaonan Xu^1^, Zhanping Liang^1^, Xuanran Feng^1^, Zhuo Wang^3^, Pu Ai^1,7^, Lu Ding^1^, Qingyuan Cai^1,8^, Yi Wang^9^, Yanyan Zhang^10^, Jie Zhu^10^, Bingbo Zhang^2,3,4*^ and Jialin C. Zheng^1,4,5,6*^*

^1^Center for Translational Neurodegeneration and Regenerative Therapy, Tongji Hospital affiliated to Tongji University School of Medicine, Shanghai 200065, China. ^2^The Institute for Translational Nanomedicine, Shanghai East Hospital, Shanghai 200120, China. ^3^The Institute for Biomedical Engineering & Nano Science, School of Medicine, Tongji University, Shanghai 200092, China. ^4^Shanghai Frontiers Science Center of Nanocatalytic Medicine, Tongji University School of Medicine, Shanghai 200331, China. ^5^Translational Research Institute of Brain and Brain-Like Intelligence, Shanghai Fourth People's Hospital affiliated to Tongji University School of Medicine, Shanghai 200434, China. ^6^Key Laboratory of Spine and Spinal cord Injury Repair and Regeneration (Tongji University), Ministry of Education, Shanghai 200065, China. ^7^Wuxi Clinical College of Anhui Medical University, Hefei 230022, China. ^8^Franklin & Marshall College, PA, 17603, United States. ^9^Center for Translational Neurodegeneration and Regenerative Therapy, Yangzhi Rehabilitation Hospital affiliated to Tongji University, Shanghai 200065, China. ^10^Center for Translational Neurodegeneration and Regenerative Therapy, Shanghai Tenth People’s Hospital affiliated to Tongji University School of Medicine, Shanghai 200072, China.

^ǂ^These authors contributed equally to this work

*Corresponding authors: Drs. Xiaohuan Xia, Bingbo Zhang, and Jialin C. Zheng. Email: [xiaohuan_xia1@163.com](mailto:xiaohuan_xia1@163.com); [bingbozhang@tongji.edu.cn](mailto:bingbozhang@tongji.edu.cn); [jialinzheng@tongji.edu.cn](mailto:jialinzheng@tongji.edu.cn)


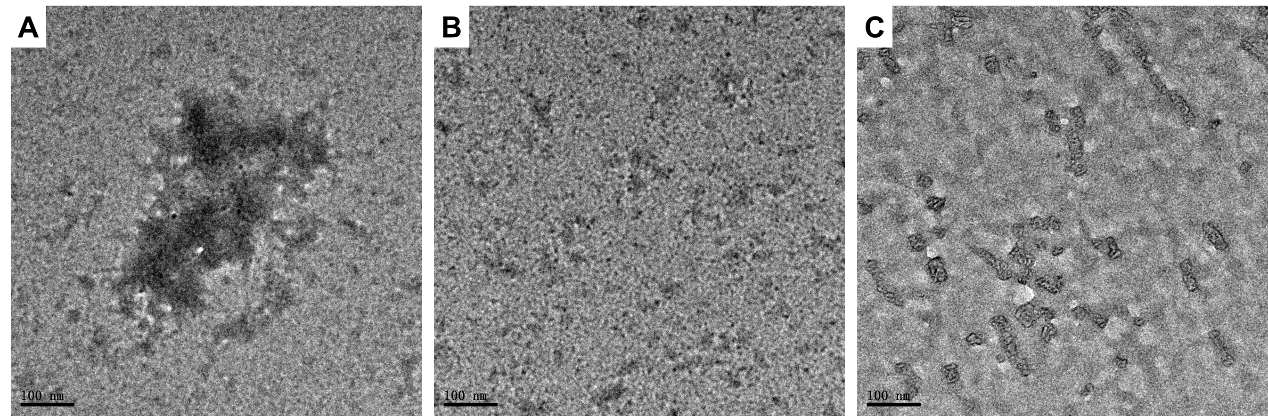


**Figure S1.** TEM images of Pd@insulin synthesized by different ratio of protein to Pd^2+^ concentration (**A**) insufficiency (**B**) proper and (**C**) excessive amounts of insulin. The concrete ratio was listed in Supplementary table 1. (Scale bar 100 nm)


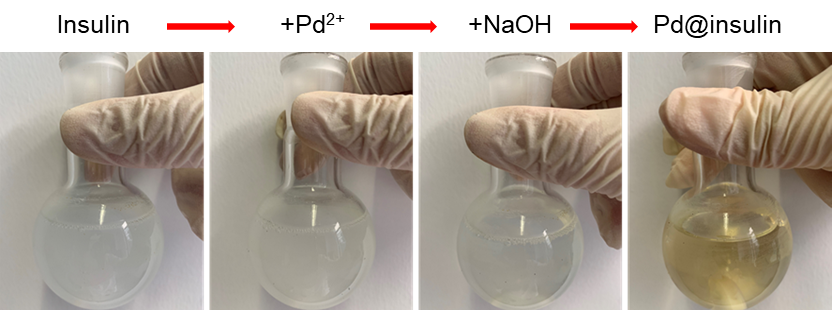


**Figure S2.** The solution variation from insulin to Pd@insulin clusters *via* the protein-incubated method.


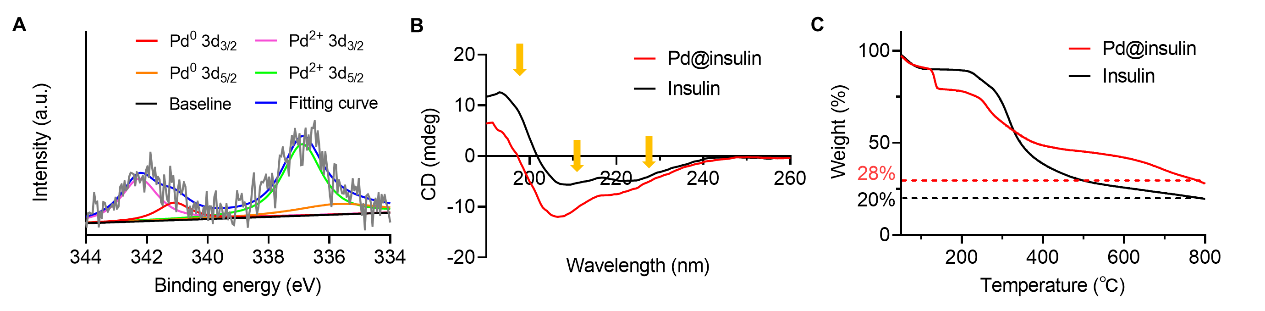


**Figure S3.** (**A**) XPS analysis of the Pd peak of Pd@insulin clusters. (**B**) CD spectrum (arrows point out the peaks of α-helix) and (**C**) Thermo gravimetric analyses (TGA) of Pd@insulin clusters and insulin.

**
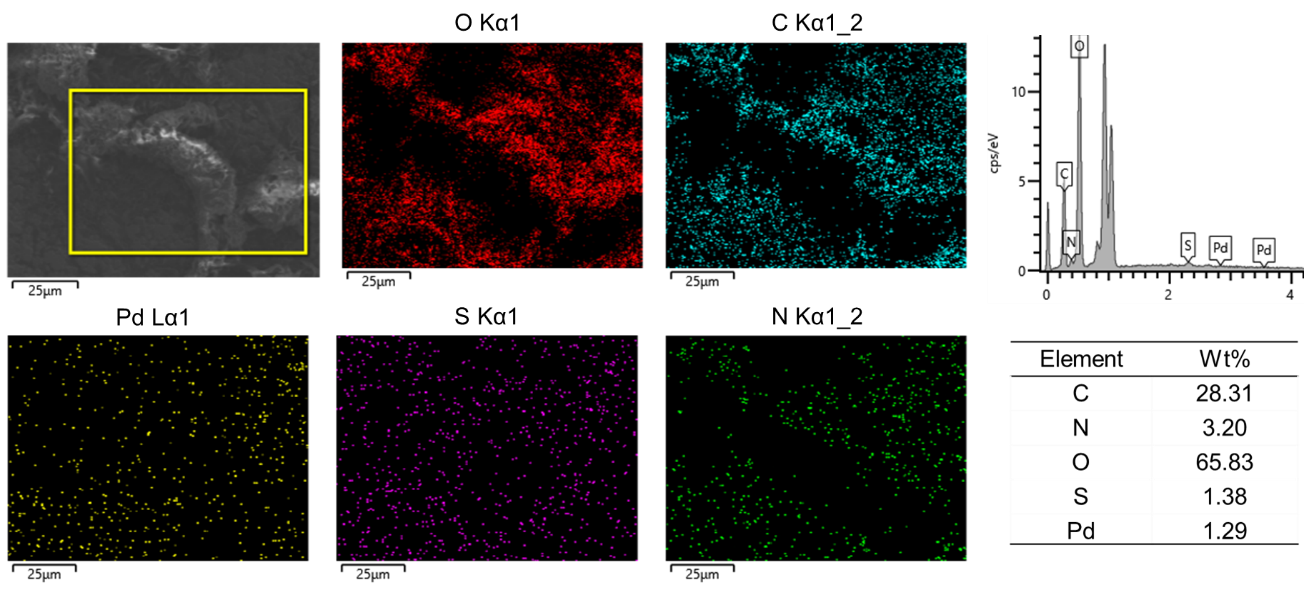
**

**Figure S4.** SEM image of Pd@insulin powder, with element mapping and EDX analyses.


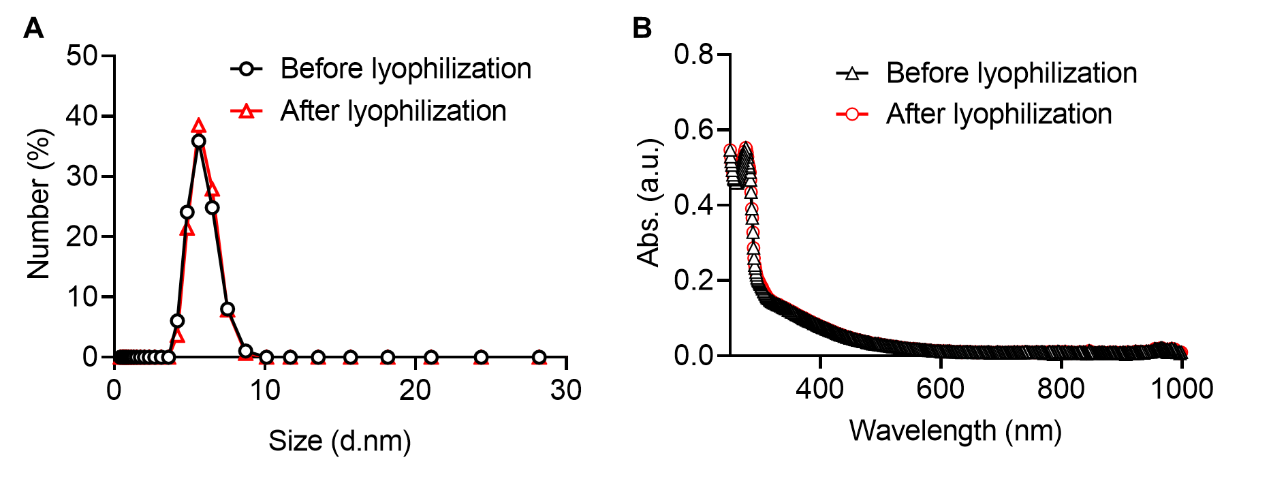


**Figure S5.** (**A**) DLS and (**B**) UV-vis analyses of Pd@insulin before and after lyophilization.

**Figure S6.** DLS size variation of Pd@insulin re-dissolved in double distilled water (ddWater), PBS, DMEM and saline (n = 3).


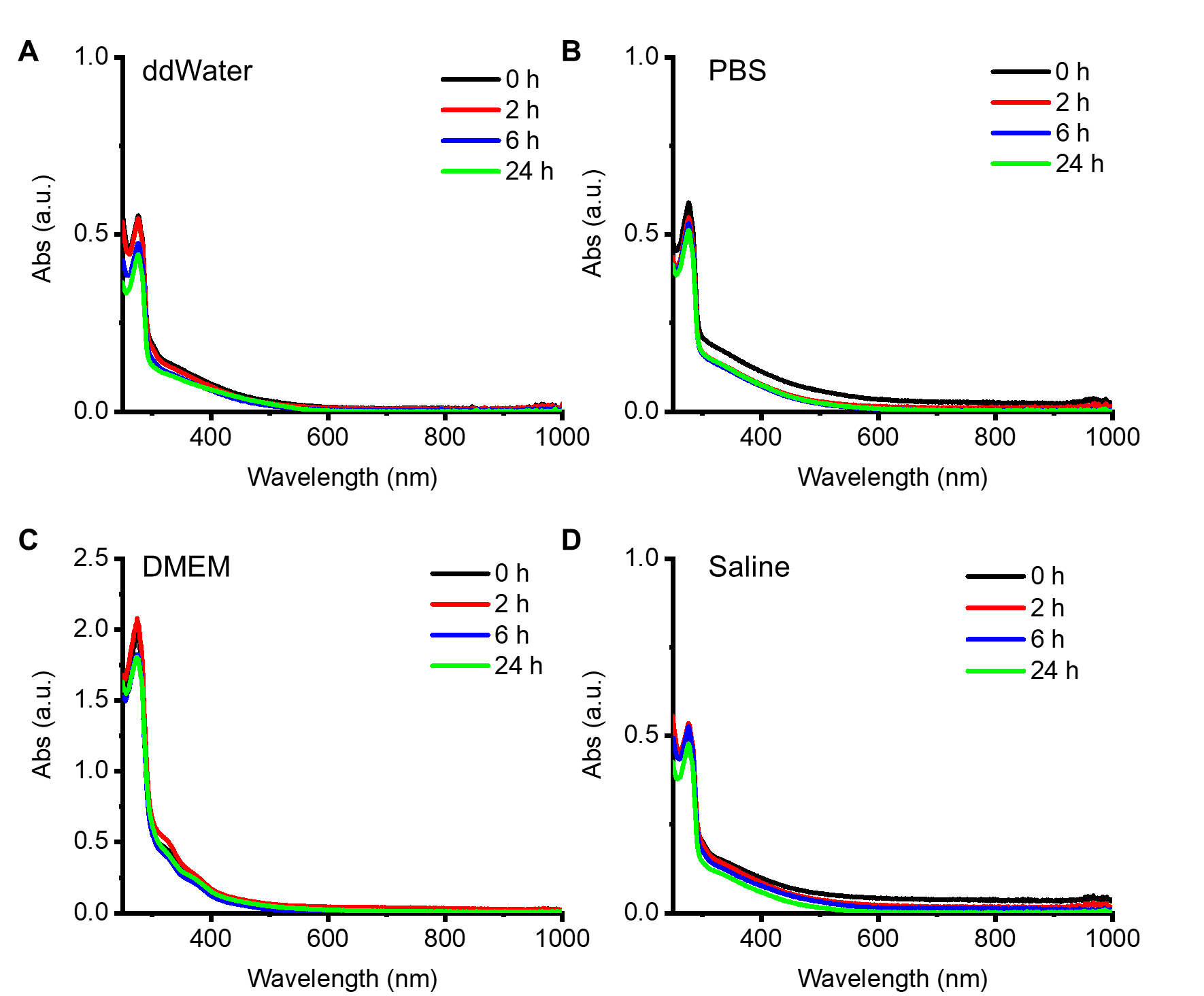


**Figure S7.** UV-vis spectrum of Pd@insulin re-dissolved in (**A**) ddWater (**B**) PBS (**C**) DMEM and (**D**) saline.


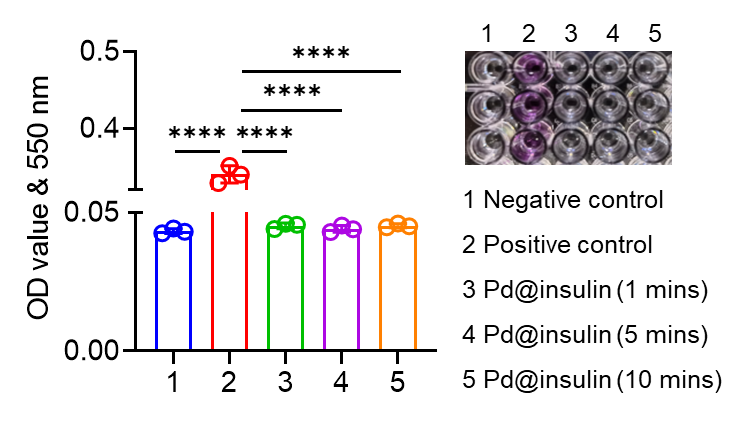


**Figure S8.** The fate of H_2_O_2_ evaluation after Pd@insulin nanoclusters treatment with different times *via* Griess reagent (Strong absorbance at 550 nm indicate the free radicals’ formation). Data are all shown as mean ± SD. Statistical analysis of was performed by one-way ANOVA with a Tukey post hoc test.

**Figure S9.** Multiple rounds ROS scavenging evaluation for Pd@insulin (result indicates no performance loss of Pd@insulin nanocluster in ROS scavenging reaction). Data are all shown as mean ± SD. Statistical analysis of was performed by unpaired Student’s t-test.


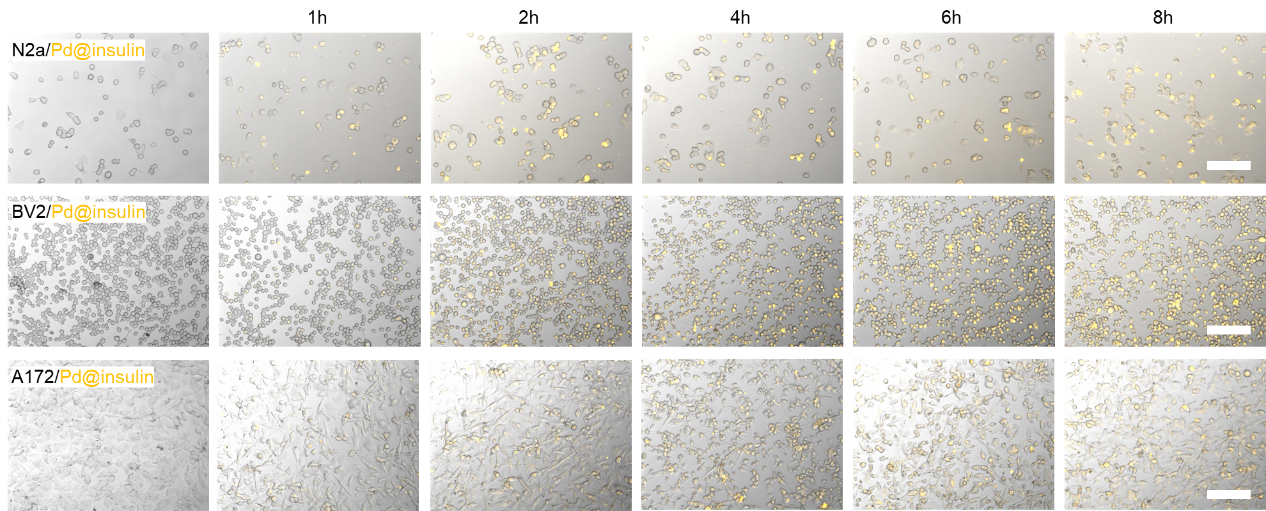


**Figure S10.** N2a, BV2 and A172 cell lines intake efficiency of Cy3-labeled Pd@insulin at different time point (Scale bar 200 μm).


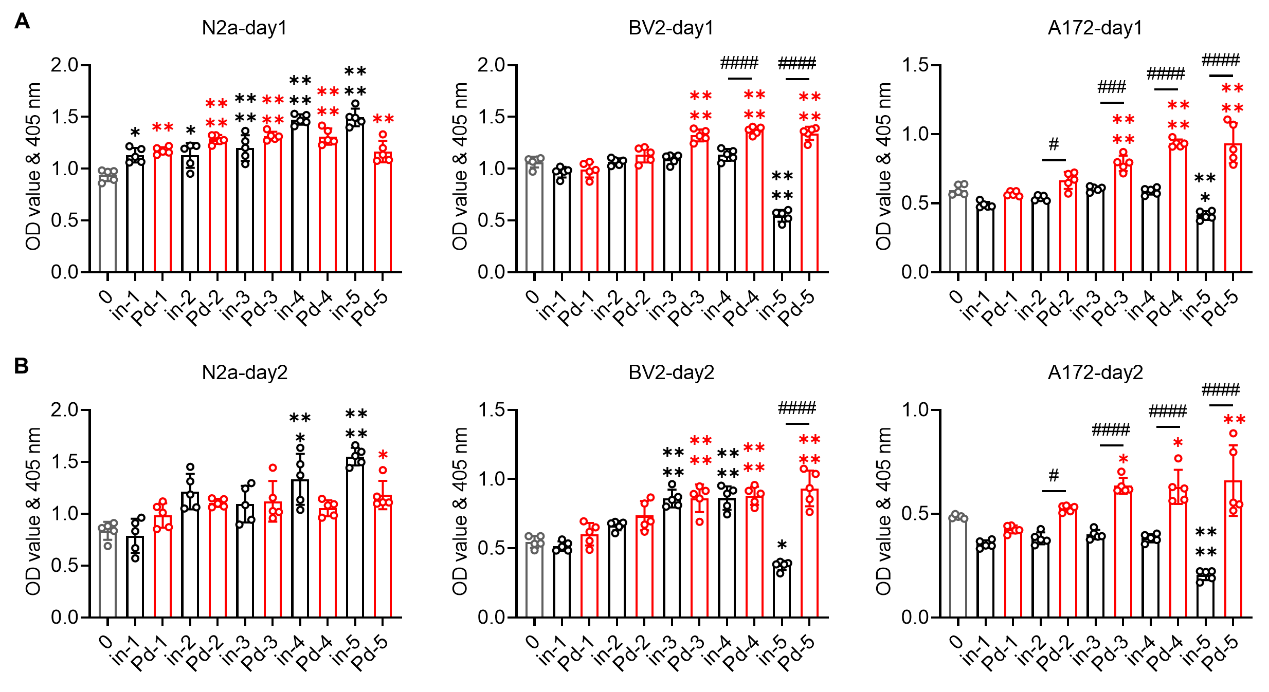


**Figure S11.** *In vitro* cytotoxicity of Pd@insulin clusters towards N2a, BV2 and A172 cells for (**A**) 24 h and (**B**) 48 h (n = 5, * represents the significant difference between Ctrl, # represents the significant difference between Pd@insulin and insulin) (n = 5). Data are all shown as mean ± SD. Statistical analysis of was performed by one-way ANOVA with a Tukey post hoc test.

**
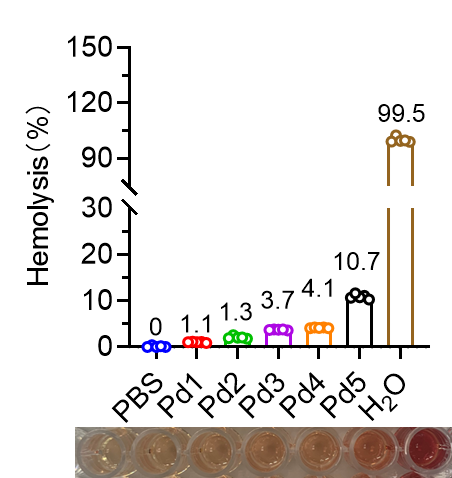
**

**Figure S12.** Hemolysis evaluation of Pd@insulin with different concentration (n = 5). Data are all shown as mean ± SD.

**Figure S13.** The ability of Pd@insulin (blue arrowhead) to reduce blood glucose equivalently to 14% pure insulin solution (n = 3), the blood glucose detect was post Pd@insulin treatment for 1 h. Data are all shown as mean ± SD.


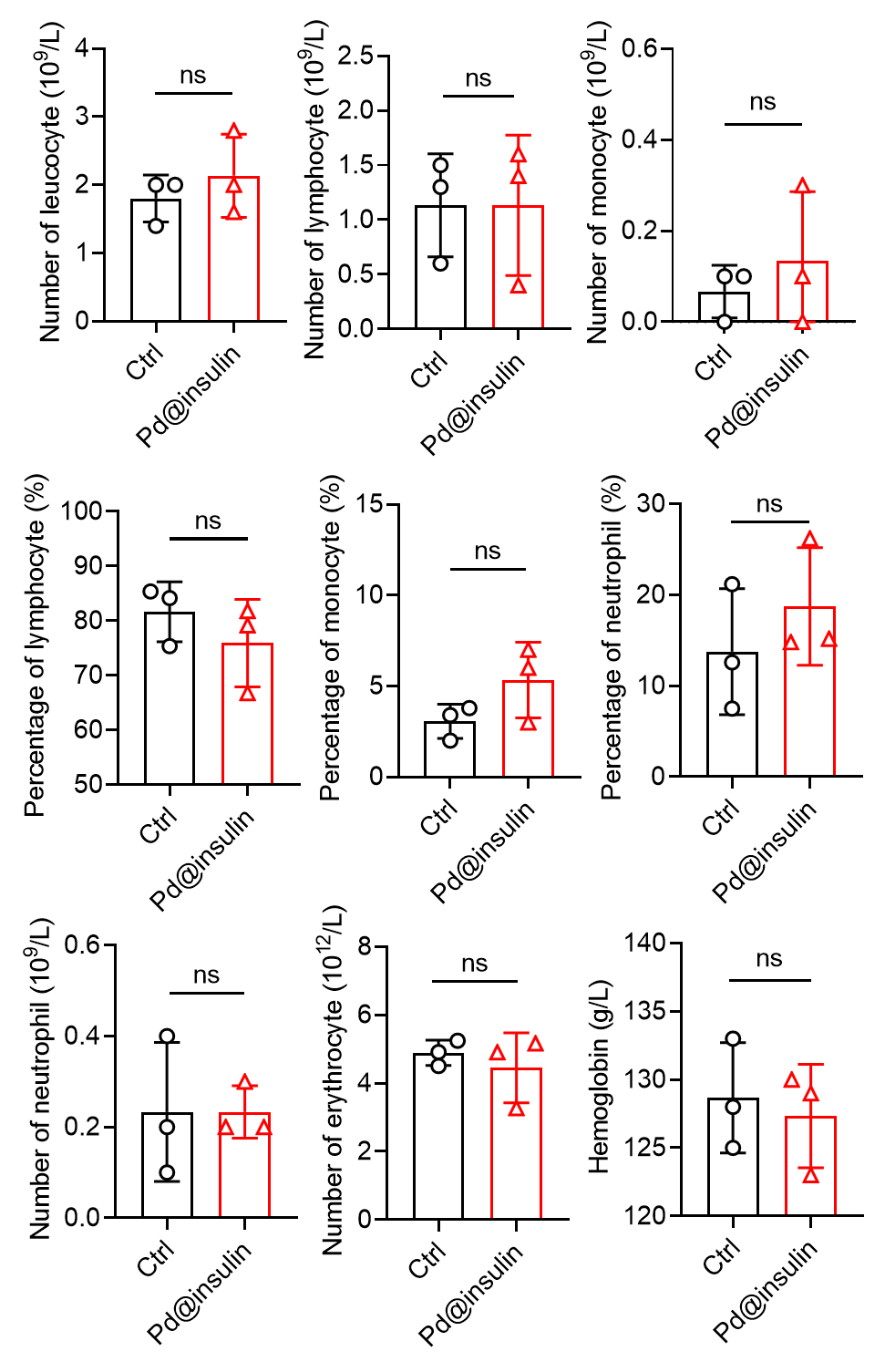


**Figure S14.** Serum biochemistry between normal mice (Ctrl) and Pd@insulin-treated mice (n = 3). Data are all shown as mean ± SD. Statistical analysis was performed by unpaired Student’s t-test.


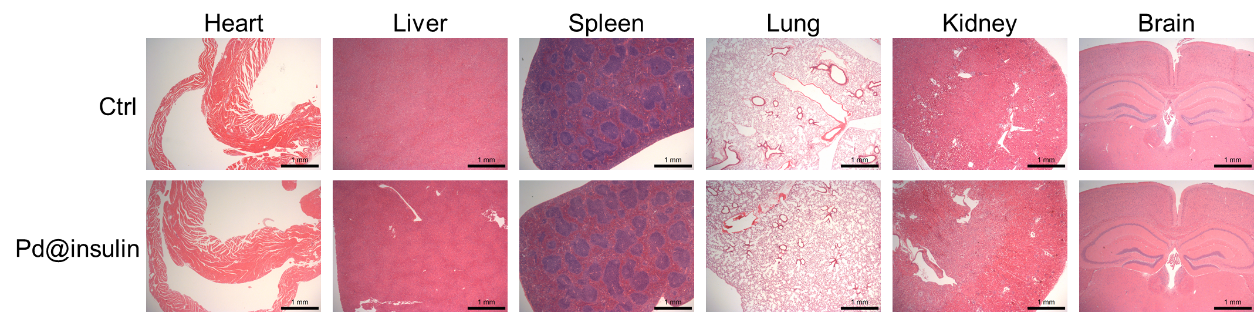


**Figure S15.** Representative H&E staining of mice main tissues post Pd@insulin intravenous injection for successive 6 days (Scale bar 1 mm).


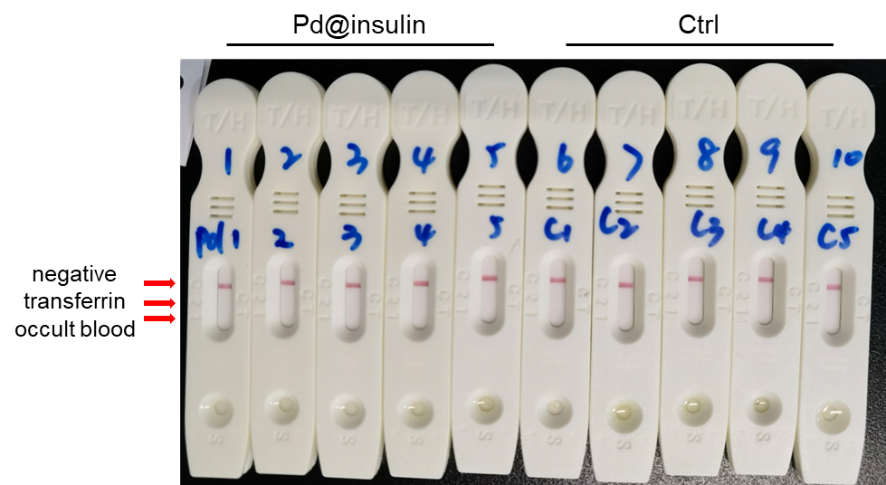


**Figure S16.** Feces examination from normal mice (Ctrl) and Pd@insulin-treated mice (1 indicates occult blood positive, 2 indicates transferrin positive, and C indicates both negative) (n = 5).


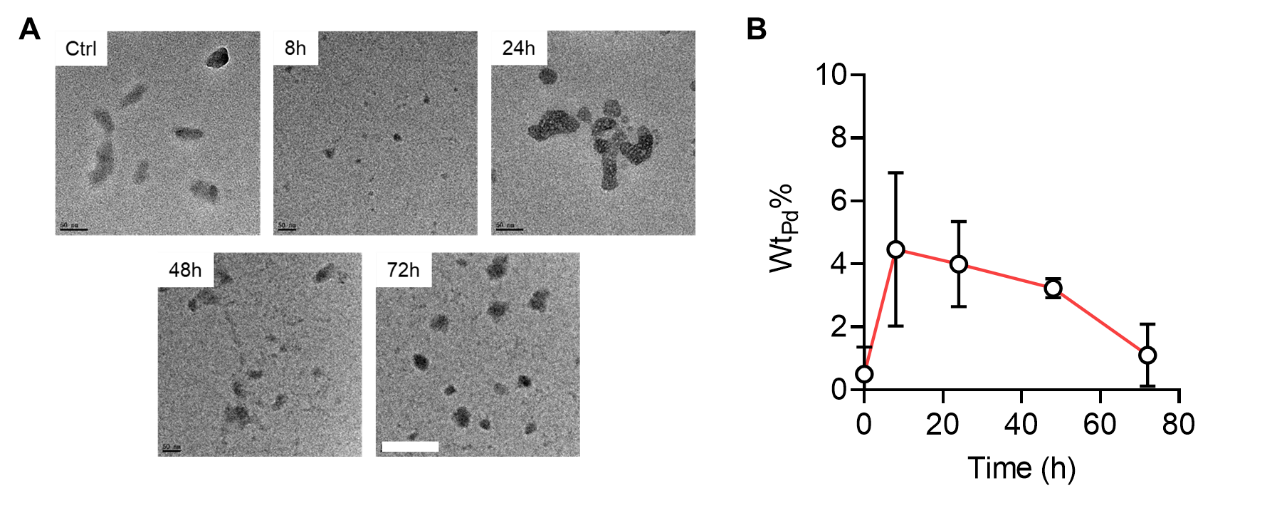


**Figure S17.** (**A**) TEM images of mice urine at different time points post Pd@insulin intravenous injection (Scale bar 100 nm) and (**B**) Pd weight ratio by EDX analysis. Data are all shown as mean ± SD.


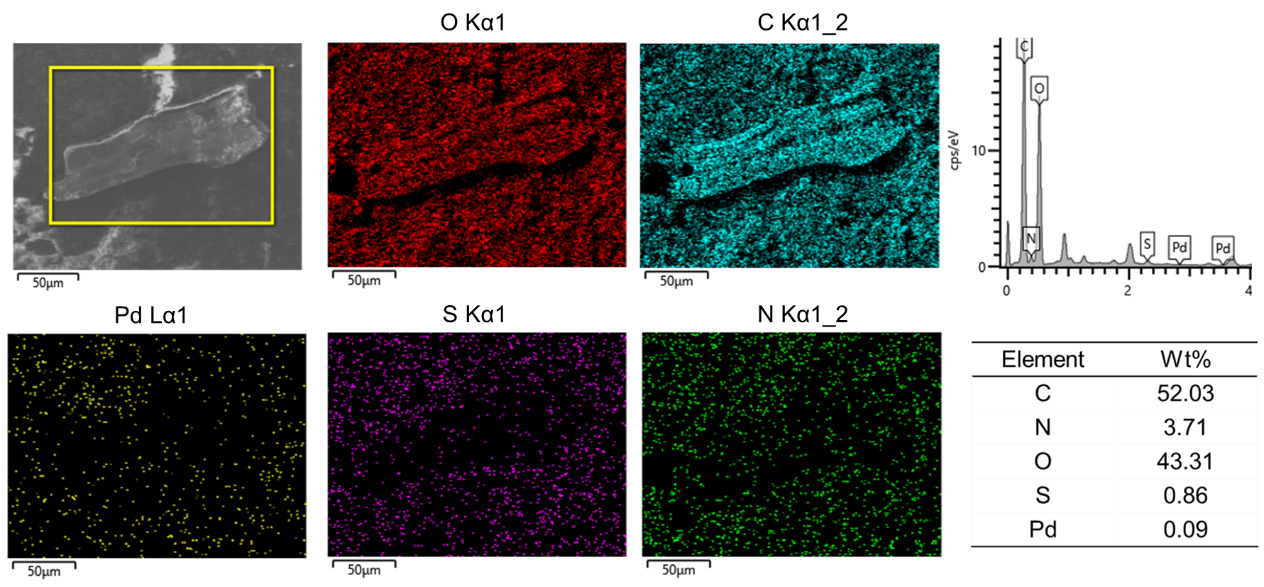


**Figure S18.** SEM image with mapping and EDX analysis of the fracture surface of mice feces at 24 h post Pd@insulin intravenous injection.


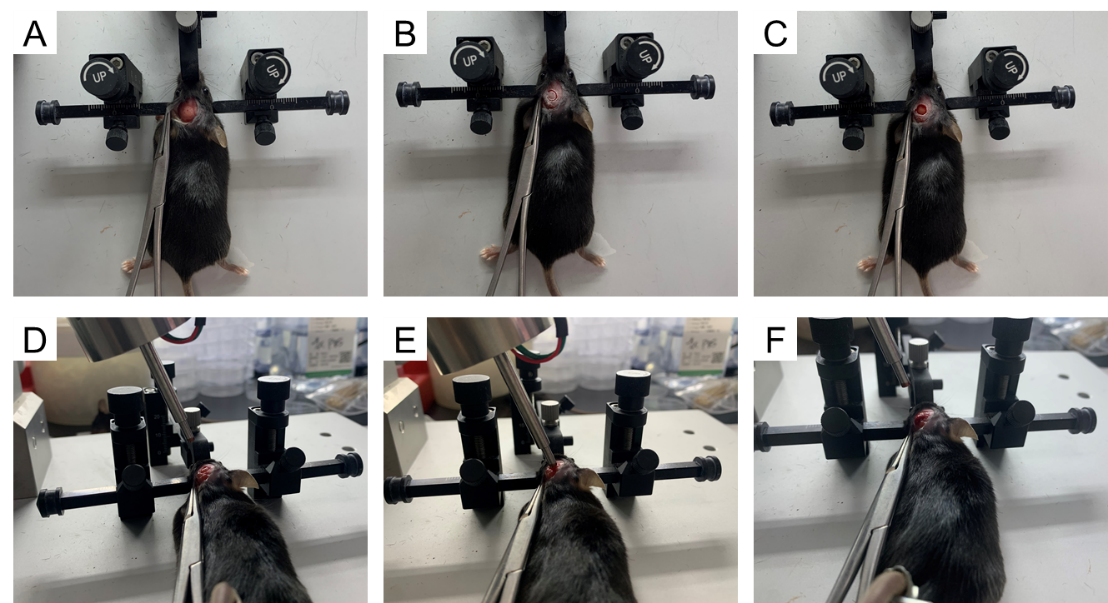


**Figure S19.** Mice TBI processes. (**A**) Fixation and scalp open (**B**) and (**C**) craniotomy (**D**) and (**E**) impacting piston location (**F**), traumatic impact.


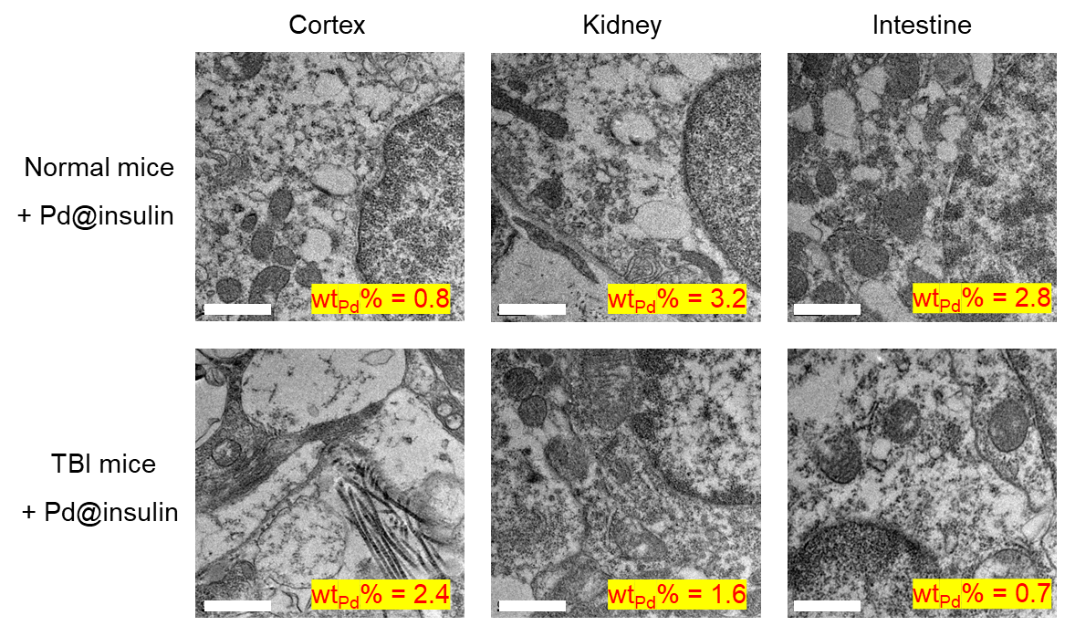


**Figure S20.** TEM images of the cortex, kidney and intestine from normal and TBI mice, with EDX analysis, post Pd@insulin intravenous injection (Scale bar 1 μm).


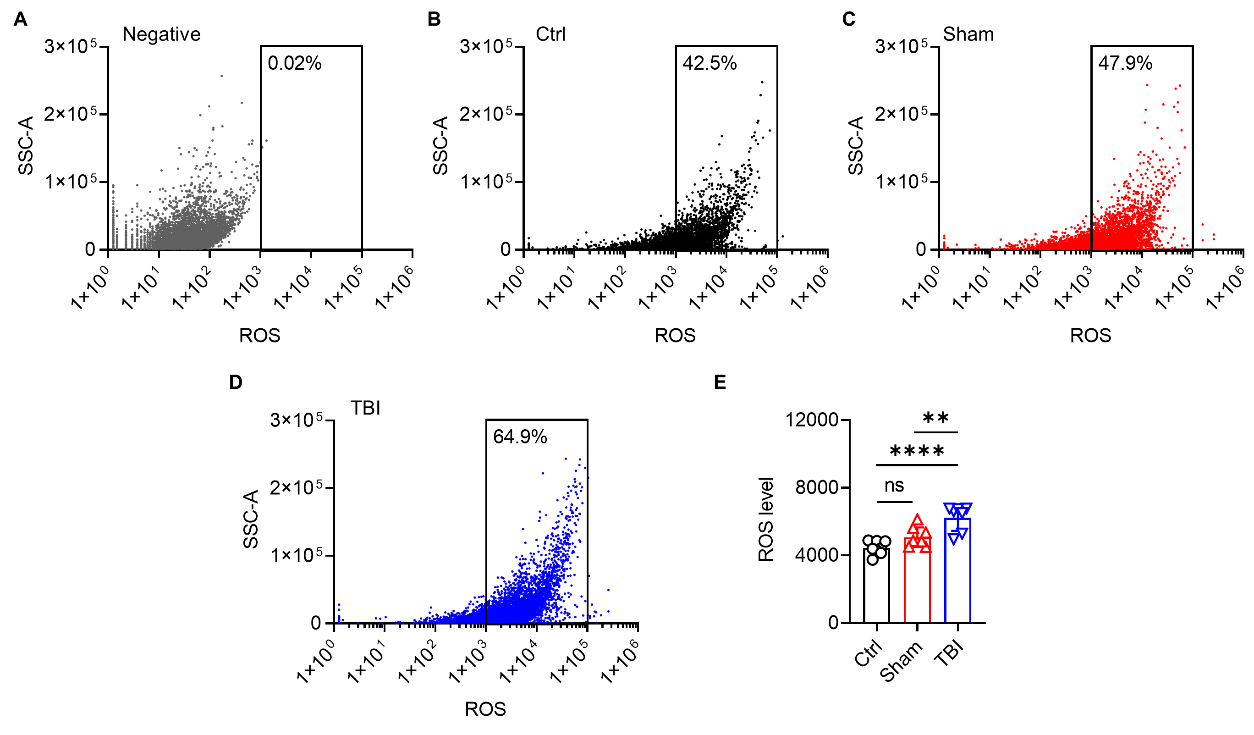


**Figure S21.** Total ROS level of mice brain. Flow cytometry analysis of ROS (10,000 cells per group) for (**A**) negative (**B**) naïve (Ctrl) (**C**) Sham and (**D**) TBI mice, and (**E**) quantitative analysis of ROS level from each mouse (n = 7-8). Data are all shown as mean ± SD. Statistical analysis was performed by one-way ANOVA with a Tukey post hoc test.


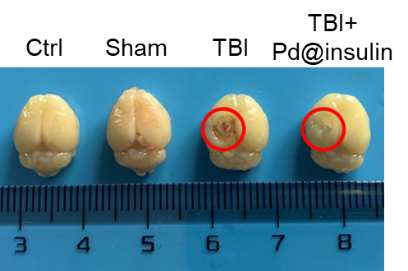


**Figure S22.** Representative image of mice brains, from Ctrl, Sham, TBI, and TBI post successive 6-day Pd@insulin intravenous injections (The red circle showed injured cortex).


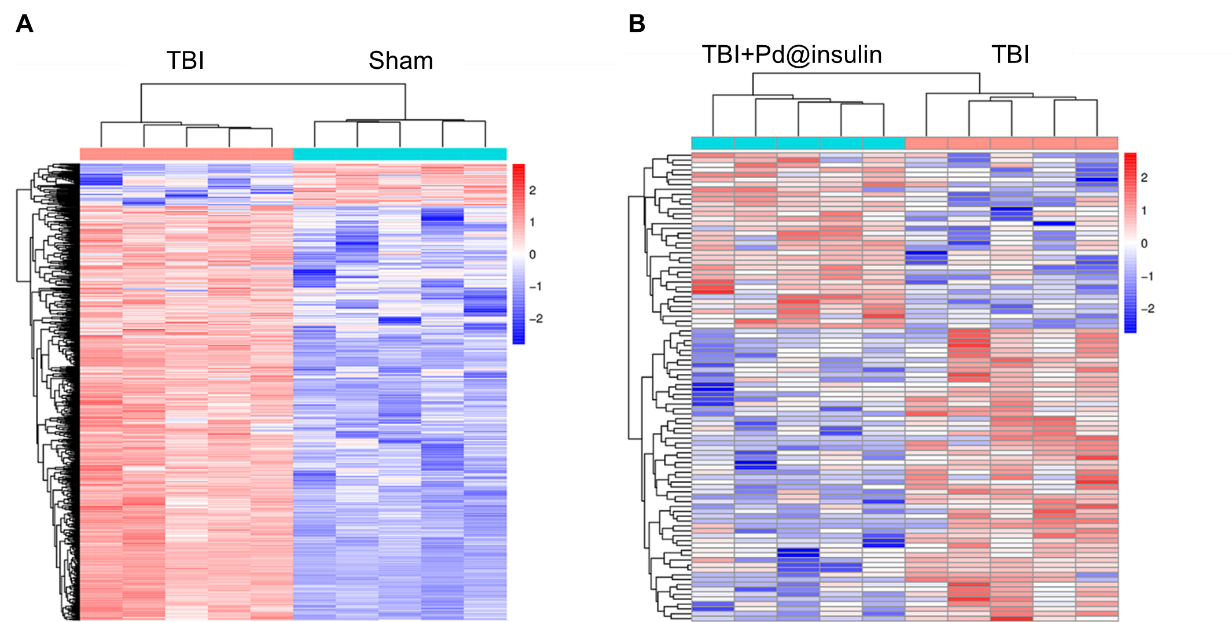


**Figure S23.** Differential genes between (**A**) TBI and Sham (**B**) TBI and TBI post Pd@insulin treatment (TBI+Pd@insulin) (p < 0.05, fold change > 1.5, n = 5).

**Table S1.** The exploration of the ratio of insulin to Pd^2+^ for Pd@insulin synthesis

|  | ddW(mL) | Insulin powder (mg) | 100 mM Na_2_PdCl_4_ (μL) |
| --- | --- | --- | --- |
| 1 | 50 | 30 | 50 |
| 2 | 50 | 30 | 25 |
| 3 | 50 | 30 | 5 |

**Table S2.** The drug concentration for treating cells

|  | Pd^2+^ concentration (μg/mL) | Pd@insulin concentration (mg/mL) |  | Insulin concentration (mg/50mL) |
| --- | --- | --- | --- | --- |
| Pd-1 | 1.25 | 0.3 | in-1 | 7.5 |
| Pd-2 | 2.5 | 0.6 | in-2 | 15 |
| Pd-3 | 5 | 1.2 | in-3 | 30 |
| Pd-4 | 10 | 2.4 | in-4 | 60 |
| Pd-5 | 20 | 4.8 | in-5 | 120 |

**Table S3.** Mice primers sequences for qPCR

| Gene name | Forward (5’-3’) | Reverse (5’-3’) |
| --- | --- | --- |
| *Oasl2* | GAAGGGTTCGTAGCCGTGAT | CCACCTGTTCCCATCCCTTT |
| *Rtp4* | GAATGCTGTTCCCCGATGACT | CCTGAGCAGAGGTCCAACTTC |
| *Glycam1* | TCAGTCTTGCTGCCACCTC | CTCTCCTCACTGGTGTAGCTG |
| *Ifi47* | TGAATCCGCTGATGTTGGGA | GTCTGCGTGGAAATTGGGTG |
| *Slfn4*  *Arg1*  *TNFα*  *IL1β* | GAGGAGGACACACAACCCG  AGACAGCAGAGGAGGTGAAGAG  TACTGAACTTCGGGGTGATTGGTCC  CCAGCAGGTTATCATCATCATCC | TCCCCTGGACACTTGACTTG  CGAAGCAAGCCAAGGTTAAAGC  CAGCCTTGTCCCTTGAAGAGAACC  CTCGCAGCAGCACATCAAC |
| *Sirt1* | GGCCGCGGATAGGTCCAT | CCGCAAGGCGAGCATAGATA |
| *Gapdh* | CATGTTCCAGTATGACTCCACTC | GGCCTCACCCCATTTGATGT |

**Video S1.** Balance beam test for mice motor ability test


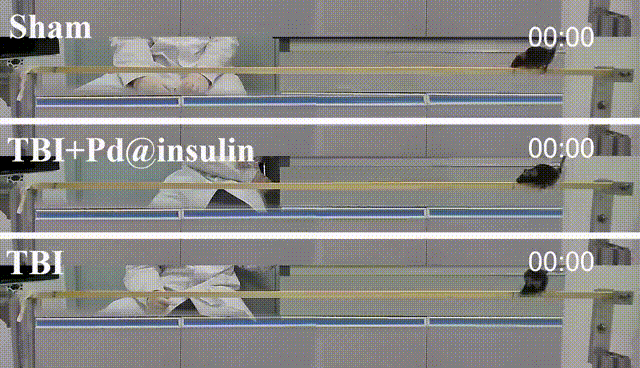


**Video S2.** Y maze test for mice cognition and spatial memory test


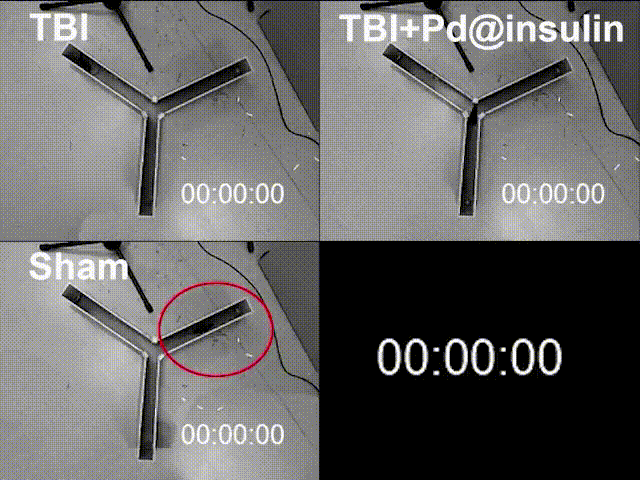

Supplement: Supplementary file 1 — Additional file 1: Figure S1. TEM images of Pd@insulin synthesized by different ratio of protein to Pd2+ concentration (A) insufficiency (B) proper and (C) excessive amounts of insulin. The concrete ratio was listed in Supplementary table 1. (Scale bar 100 nm). Figure S2. The solution variation from insulin to Pd@insulin clusters via the protein-incubated method. Figure S3. (A) XPS analysis of the Pd peak of Pd@insulin clusters. (B) CD spectrum (arrows point out the peaks of α-helix) and (C) Thermo gravimetric analyses (TGA) of Pd@insulin clusters and insulin. Figure S4. SEM image of Pd@insulin powder, with element mapping and EDX analyses. Figure S5. (A) DLS and (B) UV–vis analyses of Pd@insulin before and after lyophilization. Figure S6. DLS size variation of Pd@insulin re-dissolved in double distilled water (ddWater), PBS, DMEM and saline (n = 3). Figure S7. UV–vis spectrum of Pd@insulin re-dissolved in (A) ddWater (B) PBS (C) DMEM and (D) saline. Figure S8. The fate of H2O2 evaluation after Pd@insulin nanoclusters treatment with different times via Griess reagent (Strong absorbance at 550 nm indicate the free radicals’ formation). Data are all shown as mean ± SD. Statistical analysis of was performed by one-way ANOVA with a Tukey post hoc test. Figure S9. Multiple rounds ROS scavenging evaluation for Pd@insulin (result indicates no performance loss of Pd@insulin nanocluster in ROS scavenging reaction). Data are all shown as mean ± SD. Statistical analysis of was performed by unpaired Student’s t-test. Figure S10. N2a, BV2 and A172 cell lines intake efficiency of Cy3-labeled Pd@insulin at different time point (Scale bar 200 μm). Figure S11. In vitro cytotoxicity of Pd@insulin clusters towards N2a, BV2, and A172 cells for (A) 24 h and (B) 48 h (n = 5, * represents the significant difference between Ctrl, # represents the significant difference between Pd@insulin and insulin) (n = 5). Data are all shown as mean ± SD. Statistical analysis of was performed by one-way [file 12951_2022_1495_MOESM1_ESM.docx]
